# Bioactive Compounds from Mexican Varieties of the Common Bean (*Phaseolus vulgaris*): Implications for Health

**DOI:** 10.3390/molecules22081360

**Published:** 2017-08-17

**Authors:** Celia Chávez-Mendoza, Esteban Sánchez

**Affiliations:** Coordinación en Tecnología de Productos Hortofrutícolas y Lácteos, Centro de Investigación en Alimentación y Desarrollo A. C., Avenida Cuarta Sur No. 3820 Fraccionamiento Vencedores del Desierto. Cd. Delicias, Chihuahua C.P. 33089, Mexico; esteban@ciad.mx

**Keywords:** *Phaseolus vulgaris*, nutraceutical compounds, effect on health, common Mexican bean

## Abstract

As Mexico is located within Mesoamerica, it is considered the site where the bean plant originated and where it was domesticated. Beans have been an integral part of the Mexican diet for thousands of years. Within the country, there are a number of genotypes possessing highly diverse physical and chemical properties. This review describes the major bioactive compounds contained on the Mexican varieties of the common bean. A brief analysis is carried out regarding the benefits they have on health. The effect of seed coat color on the nutraceutical compounds content is distinguished, where black bean stands out because it is high content of anthocyanins, polyphenols and flavonoids such as quercetin. This confers black bean with an elevated antioxidant capacity. The most prominent genotypes within this group are the “Negro San Luis”, “Negro 8025” and “Negro Jamapa” varieties. Conversely, the analyzed evidence shows that more studies are needed in order to expand our knowledge on the nutraceutical quality of the Mexican bean genotypes, either grown or wild-type, as well as their impact on health in order to be used in genetic improvement programs or as a strategy to encourage their consumption. The latter is based on the high potential it has for health preservation and disease prevention.

## 1. Introduction

The common bean predominates among the most produced and consumed legumes in Africa, India, Latin America and Mexico. The latter is the site where 47 of the 52 classified species of the *Phaseolus* genus were originated [1]. Although historically this food has been the main protein source in developing countries, its consumption has been decreasing during the last decades as the population has adopted a western life-style. Its consumption has been undervalued in North America and the north of Europe [2]. The same trend is observed in the countries along the Mediterranean Sea and this has led to an increased incidence of chronic diseases such as cancer, obesity and cardiovascular diseases [3]. According to the World Health Organization (WHO) [4], the latter is the most common cause of death caused by non-transmissible diseases (NTD), summing up to 17.7 million cases each year, followed by cancer (8.8 million cases), respiratory diseases (3.9 million cases) and diabetes (1.6 million cases). These four groups of diseases are responsible for 81% of the deaths caused by NTD. An increasing amount of scientific evidence currently supports the role of bioactive compounds on disease prevention and the treatment because of their beneficial effect on health [5]. Several studies report that common bean is a food with a high content of proteins, carbohydrates, diet fiber, minerals and vitamins [6]. Moreover, they contain several bioactive compounds that do not only confer a color to the seed, such as flavonol glycosides, anthocyanins and condensed tannins (proanthocyanidins), but they also possess biological activity [7]. Some clinical studies have demonstrated a beneficial effect from bean consumption on glycemic index and a protective role against the establishment of type 2 diabetes because of their high polyphenol content that confer them with a significant antioxidant effect. Furthermore, several lines of evidence suggest that beans consumption decreases the risk of ischemic heart and cardiovascular diseases, stomach and prostate cancer, weight control and obesity, stress attenuation, anxiety and depression in elderly populations, among other roles [2,8].

Bean consumption has been an integral part of the diet in Mexico for thousands of years [9]. The studies conducted on this subject have identified that the site where bean was originated and domesticated is Mesoamerica, in particular west and south of Mexico. From those sites, the species migrated towards South America [10]. Currently, a great genetic diversity of beans is being grown in Mexico, including the wild-type varieties that still remain without classification. There are three among the most prominent local genetic races: Durango, Jalisco and Mesoamerica. There is yet another that has been introduced: Nueva Granada. These grains are sowed all along the country, in different times during the year and by using several production systems. All of this contributes to its genetic diversity. At the northeast region of Mexico, the predominant variant are pale yellow-colored beans (“Peruano”, “Azufrado”, “Mayocoba”), whereas at the north-center the “Pinto”, “Bayo”, “Flor de Mayo”, “Garbancillo” and “Negro” varieties are the most common. At the central region of the country, several local bean types predominate, such as “Flor de Mayo”, “Flor de Junio”, “Negro” and some “Criollo” varieties, whereas small-grain beans are the most important along with those black and cloudy in the humid tropic regions. The extent of industrialization of this food is still low, with barely a 5% participation from the industrial sector [11]. The commercial varieties that are currently produced are consumed either cooked or fried [12].

Several studies have quantified phytochemicals contained on several varieties of the common Mexican bean. Thus, total phenols, anthocyanins, tannins, flavonoids, lectins, phytic acid, oligosaccharides, and other bioactive compounds were identified as the most predominant [12,13,14,15,16,17]. Additionally, it has been demonstrated that consumption of some varieties of the common Mexican bean decreases carcinogenic tumor occurrence in Sprague-Dawley rats [18].

Culture enhancement is promoted by extending the genetic basis and the knowledge on available resources. In some countries as Italy and Spain some studies have been carried out in order to collect, to know and to protect their bean races that were previously introduced from America approximately five centuries ago. Regarding Mexico, the fields are currently in a state of abandonment and the lack of culture continuity is causing the loss of information on their benefits as well as the handling of the traditionally cultured varieties. This may also lead to the loss of the varieties themselves. The knowledge regarding the genetic diversity occurring in their cultivars will contribute to expand the basis for improvement programs and it may contribute to the efforts carried out in order to increase bean per capita consumption while decreasing fast food consumption [19].

The aim of this review is to reveal the nutrient composition and the bioactive compound content of the common Mexican bean varieties and their possible role in consumer’s health.

## 2. Functional Food and Nutraceutical/Bioactive Compounds

To some extent, all foods are functional as they provide flavor, odor and nutritional value. However, currently consumed food is being intensively analyzed in order to discover its physiologic benefits as it may decrease the risk of chronical diseases. These research efforts have sparked a global interest on the increasing amount of foods now classified as functional [20].

A widely accepted definition for functional food is still lacking [20]. Some authors point out that strictly, this is a marketing term and it has not been accepted by legal authorities worldwide [21]. Several definitions exist for this type of food. Drago et al. [22] define them as “food products of animal or vegetal origin that are consumed on the daily diet that do not only provide nutrients but they possess bioactive compounds”. Whereas the Federal Health Department in Canada [23] indicates that they are “food with a similar appearance to a conventional food, consumed as part of a normal diet, that possess proven physiological benefits and/or they reduce the risk of chronic diseases beyond the basic nutritional functions”. Likewise, The European Commission Concerted Action on Functional Food Science in Europe (FUFOSE) [24] classifies a food as functional “if it is satisfactorily demonstrated to affect beneficially one or more target functions in the body, beyond adequate nutritional effects, in a way that is relevant to either an improved state of health and well-being and/or reduction of risk of disease”. Finally, in the United States they are defined as “food or their components that provide a benefit for health beyond basic nutrition” [25].

Apparently, there is no consensus about how this type of food is defined. However, most of the definitions agree that functional food must have health benefits beyond basic nutrition. In this regard, Nicoletti [26] points out that the only consensus that has been reached is related to the fact that they are not drug products, although this is still a confusing issue.

According to Henry [21], the “functional food” concept involves a synergy between two fundamental aspects for life: health and diet. Frequently, this termed is referred to as a new emerging field. However, the concept of functional food was first described on the ancient Vedic texts in India and on traditional Chinese medicine. The views regarding this type of food reflect the eastern philosophy that considers both food and medicine having a common origin. Such concept was first developed in Japan during the 1980s decade due to the elevated costs of health care. In those years, the Health and Welfare Ministry established a regulatory system in order to approve some food possessing well-documented benefits aiming to improve the health of the aging population [20,21]. Another term that is frequently used as synonym of functional food, although less accepted by consumers, is “nutraceutical”. It refers to a bioactive compound providing some benefit to health [20]. This term is derived from the words “nutrition” and “pharmaceutical” and it is defined as “a food (or parts from it) providing medical or health benefits, including the prevention and/or treatment of a disease” [27]. Conversely, Biesalski et al. [28] state that this term refers to a wide variety of bioactive compounds contained in foods that provide health benefits beyond a basic nutrition effect. Thus, bioactive compounds are part of functional food.

Nutraceuticals have been classified based on several criteria: food source, mechanism of action, chemical nature and specific benefit for health. They may be macronutrients (omega-3 fatty acids), micronutrients (vitamins and minerals) and phytochemicals. Thus, based on their food source, nutraceuticals may be classified as fiber diet, polyunsaturated fatty acids, probiotics, prebiotics, antioxidants, vitamins, polyphenols and spices [27]. Independently, Nasri et al. [29] reported that the term nutraceutical applies to products isolated from herbs, diet supplements (nutrients), specific diets and processed food such as cereals, soups and beverages that are used as medicines in addition to their role on nutrition. These may be used to improve health, to slow down the aging process, to prevent chronic diseases, to extend life expectancy or to support a body structure or function. Currently, these compounds are of great interest because of their high potential as nutritional agents, their therapeutic effects and their safety for consumption. Based on this, some studies have observed that the market for these products is expanding worldwide and it may reach 20 billion dollars by the end of 2018 [29].

### The Regulation for Bioactive Compounds

According to Nicoletti [26], Japan is the only country that has established a specific regulatory approval process for functional foods, whereas this category is not legally recognized in the United States. Although, according to Nasri et al. [29], in this country nutraceutical products are regulated as drug substances, food ingredients and diet supplements. The term is not equally defined in all countries and this regulation only refers to the product isolated from different food sources, that is commercialized as medicine and that is not usually linked with food. In comparison to drug products, these substances are not normally protected by a patent, although both may be used to treat or prevent diseases. Currently, the government in this country only authorizes pharmaceutical products. On the other hand, Dureja et al. [30] mention that the laws intended to regulate nutraceutical production and commercialization was approved in 1994 in the United States. This legislation, known as the Dietary Supplement Health and Education Act was issued by the FDA in order to regulate health-related products and to establish good manufacturing practices for nutraceuticals. According to Palthur et al. [31] the laws regarding nutraceutical products are complex because the concept is still not clear. In addition, in order to a regulatory policy to exist, it must be quantified and defined. However, the regulatory status of these products may differ depending on the regulation system on each country. Sarin et al. [32] point out that the FDA regulates nutraceuticals in the same manner it regulates all foods. Beforehand, it must be assured that all ingredients are safe and reliable, whereas all claims must be well-founded, true and not misleading.

## 3. The Common Bean and Its Nutritional Quality

The *Phaseolus* genus includes five domesticated species: *P. vulgaris* (common bean), *P. lunatus* (butter bean), *P. acutifolius* (tepary bean), *P. coccinetus* spp. *Coccetus* (ayocote bean) and *P. dumosus* = *P. polyanthus* (*P. coccineus* ssp. Darwinianus) (year-long bean) [10].

Mexico, as part of Mesoamerica, is considered as the site of the origin and primary domestication of several bean types. Because of its commercial value, the most prominent is the common bean *Phaseolus vulgaris*, the one that Christopher Columbus took back to Europe during the Conquest [33]. This variety was domesticated at the Tehuacán Valley (Puebla, Mexico) approximately 7000 years ago, probably along with corn. The common bean comprises two gene pools: Mesoamerican and Andean. These differ on their structure and genetic diversity levels. There are some contrasting morphological features when both groups are compared and they contribute to their differentiation. The Mesoamerican pool exhibit decreased levels of phaseolins and lectins, whereas they possess a higher content of nutritional elements, except for Fe [10].

Within the legumes category, *Phaseolus vulgaris* is the most commonly grown and consumed species in Africa, India, Mexico and several countries of Central America and South America. Currently, it is distributed among the five continents and it is an essential component of the diet. In these regions it is part of the population’s eating habits. Its consumption is mainly in the form of whole grain. However, because of its high nutritional value, its use should be diversified through its use as an ingredient to develop of new food products [34].

The bean’s nutritional properties are linked to their high protein content and, to a lesser extent, to its carbohydrate, vitamins and mineral content. Depending on the bean type, protein content ranges between 14% and 33% and its enriched in amino acids such as lysine (6.4–7.6 g/100 g protein) and phenylalanine plus tyrosine (5.3–8.2 g/100 g protein) [35]. Thus, it meets all the minimal requirements recommended by the Food and Agriculture Organization (FAO) or the World Health Organization. However, bean lacks the sulfur amino acids: methionine and cysteine [36]. A 90 g serving of beans provide 8 g of protein, almost 15% of the recommended daily consumption for an adult weighing 70 kg. The digestibility for this protein is 79% [37].

Regarding their carbohydrate content, 100 g of raw bean contain 52–76 g. The most important fraction is represented by starch [35], constituting more than 50% of the seed’s weight [36]. They are also constituted by raw fiber and less but significant amounts of mono-, di- and oligosaccharides. Beans possess slow-digestion carbohydrates and a high proportion of non-digestible carbohydrates that may be fermented in the large intestine. The non-digestible sugars that reach the colon include resistant starch, soluble and insoluble diet fiber and non-digestible oligosaccharides [37].

Within the micronutrients group, the lipid fraction is the smallest (1.5–6.2 g/100 g) and it is comprised of an acylglycerides mixture in which main fatty acids are mono- and polyunsaturated. Bean is also a good fiber source and its value ranges between 14 and 19 g/100 g raw food. Fifty percent of this is present in its soluble form. The main components of bean fiber are pectins, pentosans, hemicellulose, cellulose and lignin [35].

The chemical composition of five Mexican varieties of the common bean is presented in Table 1. The protein content was within the 15–26.35% range for the “Bayo Victoria” variety and it displayed the highest value for this nutrient. Furthermore, raw fiber ranged between 1.77 and 2.77. The latter variety also displayed the highest content. 

## 4. Bioactive Compounds on the Common Bean and Implications on Health 

Besides being an important protein, carbohydrate, fiber and mineral source, beans have been identified as rich in phytochemical compounds providing important benefits to health. Examples of such compounds are phenolic acids, flavonoids, flavan-3-ols, condensed tannins and anthocyanins. It is also rich in anti-inflammatory and antioxidant compounds that specifically protect against 2,2-diphenyl-1-picrylhydrazyl (DPPH), 3-ethylbenzothiazoline-6-sulfonic acid (ABTS) and peroxyl radicals [41]. In this section, the main bioactive compounds found on the common bean are presented. Some of their effects of human health associated with their consumption are also discussed.

### 4.1. Saponins 

**Figure 1:**
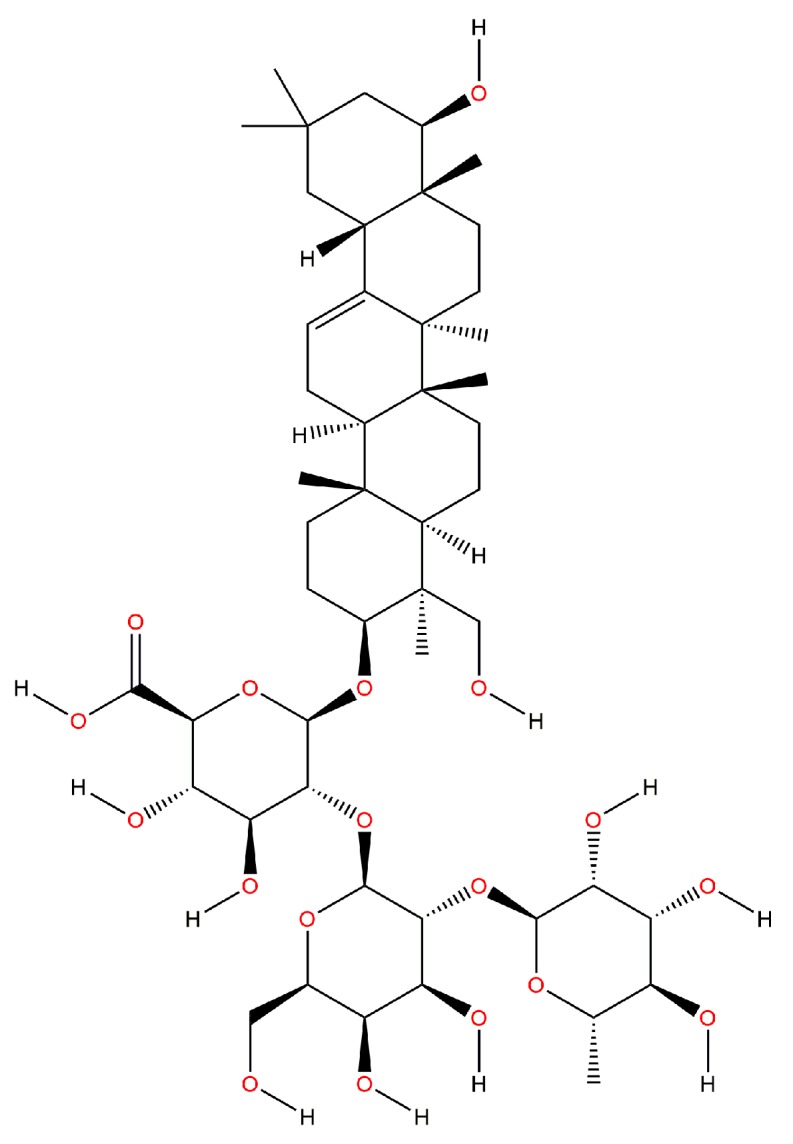
Soyasaponin I structure of *P. vulgaris* [46].

Common beans contain trace amounts of saponins. These substances are characterized by possessing a structure containing a steroidal aglycone or a triterpenoid including one or more sugar chains [42]. They are found as steroidal glycosides, steroidal alkaloid glycosides or as triterpene glycosides. Therefore, they are triterpenoids or steroids containing one or more sugar molecules on their structure [43]. Furthermore, these bioactive compounds possess a complex structure comprised by a hydrophobic steroidal nucleus and a hydrophilic moiety constituted by monosaccharide units [44]. Saponins are classified as groups A, B or E, based on their aglycones structures. Group A saponins possess glucosyl groups attached to the C-3 and C-22 positions of the aglycone, whereas those belonging to groups B and E are glycosylated only in the C-3 position. Group E saponins contain a ketone in C-22 instead of a hydroxyl group found in counterparts belonging to Group B [45]. Soyasaponin A, soyasaponin B and phaseoside I, have been reported as the main saponins contained in common bean cotyledons and seed coats, whereas other authors have found that Soyasaponin I (Figure 1) is predominant form [42].

Several epidemiological and experimental studies have demonstrated the beneficial effects of saponin on humans. It has been found that these compounds have hypocholesterolemic, immunostimulant, anti-carcinogenic, hypoglycemic, anti-thrombotic, diuretic and anti-inflammatory effects, they reduce the risk of heart diseases and they possess an elevated antioxidant capacity. It has been also reported that saponins, particularly saponin B1 from soy, have an inhibitory effect on infectivity of the human immunodeficiency virus in vitro. Additionally, a saponin-rich diet prevents tooth cavities and platelet aggregation during hypercalciuria treatment in humans. It also functions as an antidote for acute poisoning caused by lead. In addition, some epidemiological studies have shown an inverted correlation with the occurrence of kidney stones [44,47].

Table 2 shows the saponin profile of the Mexican black bean from the “Negro San Luis” variety. Its total saponin content is 42.28 mg/100 g sample and soyasaponin Af (Acetyl A2) is one of the most prominent types. 

### 4.2. Phenolic Compounds

Phenolic compounds are metabolites possessing different structure and function, and they possess an aromatic ring generally containing one or more hydroxyl groups [48]. Based on their chemical structure, they are a highly diverse group ranging from simple molecules such as phenolic acids to complex polymers such as tannins and lignin [43]. The common bean contains a great amount of polyphenols. These are bioactive compounds widely known because of their antioxidant properties, therefore they have a very important role for decreasing the risk of cardiovascular diseases, diabetes, some types of cancer, Alzheimer’s and Parkinson’s diseases. The antioxidant properties of these compounds lies on their ability to neutralize free radicals and the chelation of transition metals, thus they counteract the initiation and propagation of oxidative processes [49].

There are three main parts on legume seeds: cotyledon, seed coat and embryonic axe. The non-flavonoid phenolic compounds such as hydroxybenzoic acid and hydroxycinnamic acid are located in the cotyledon, whereas flavonoids are found in the seed coat [50].

González de Mejía et al. [51], observed that the major amounts of polyphenols compounds were located in the seed coat of the “Flor de Mayo” variety of the Mexican common bean and it represents 11% of the total seed. These authors reported that the obtained methanolic extract displayed anti-mutagenic activity against 1-nitropyrene and benzopyrene. A phenolic compound content of 145 mg/g was measured by Cardador-Martínez et al. [9] in a methanolic extract obtained from the bean seed coat of the “Flor de Mayo FM-38” variety and they also found an anti-mutagenic activity against aflatoxin B1. Furthermore, Espinosa-Alonso et al. [14] studied 62 Mexican lines of the common wild bean and they reported that total phenolic compound content was 0.90–2.11 mg gallic acid equivalent (GAE)/g of bean flour. In this study, the Mexican “Negro Jamapa” and “Frijol Pinto” varieties were also analyzed. The overall phenol concentrations were 1.41 and 1.98 mg GAE/g bean flour, respectively. The results measured on the Mexican bean lines were similar to those observed for the Vaccinium wild berries, one of the most important polyphenol source in fruits (0.81–1.70 mg GAE/g). In yet another study, Almanza–Aguilera et al. [12] analyzed 16 genotypes of the black Mexican bean and reported a total phenol range of 503.2–1062 mg/100 g of raw beans and 210–711 mg/100 g of cooked beans. They demonstrated a high content of phenolic compounds on the raw seed that were invariably reduced after the cooking (50%) and frying (64%) processes, although this decrease was different among genotypes. In some cultivars, such as that of “Negro Guanajuato” the decrease induced by cooking was minimal, whereas others such as “Negro Otomí” and “Negro San Luis” contained less phenolic compounds after being fried. However, other varieties as “Negro 8025” exhibited a higher concentration of phenolic compounds after the latter treatment. Boateng et al. [52] also reported a higher phenolic content of beans possessing a darker seed coatin comparison with those characterized by light colors. These results were confirmed by the study conducted by Orak et al. [53], in which the phenolic content of 10 varieties of the white-colored bean from Turkey was assessed resulting in decreased levels in comparison with those characterized by red and black colors.

Other factors affecting the phenolic compound content of the common bean are the storage conditions for the seeds. Mujica et al. [54] reported a significant decrease of both phenolic compounds content and antioxidant activity on Mexican common bean samples stored at 37 °C and 75% RH for 120 days when compared to those stored at 5 °C and 34% RH for 120 d. Herrera et al. [55] identified a significant effect of location, genotype and humidity treatment on the overall soluble phenolic compounds level in eight bean genotypes from the Jalisco race (most of them pink-colored mottled with cream-colored dots matching the “Flor de Mayo Anita”, “Flor de Mayo Noura”, “Flor de Junio Marcela” and “Flor de Junio Bajío” varieties), the Durango race (light cream-colored seeds with brown- or beige-colored dots of the “Pinto Zapata” and “Pinto Saltillo” varieties) and the yellow-colored Nueva Granada race (“Azufrado 26” and “Azufrado Noroeste”). They were grown in two contrasting regions (Celaya, Guanajuato and Ahome, Sinaloa, both in Mexico). The authors mention that those varieties from the Jalisco race produced with irrigation and terminal hydric stress exhibited higher total soluble phenols when compared to the Durango and Nueva Granada races.

#### 4.2.1. Flavonoids

**Figure 2:**
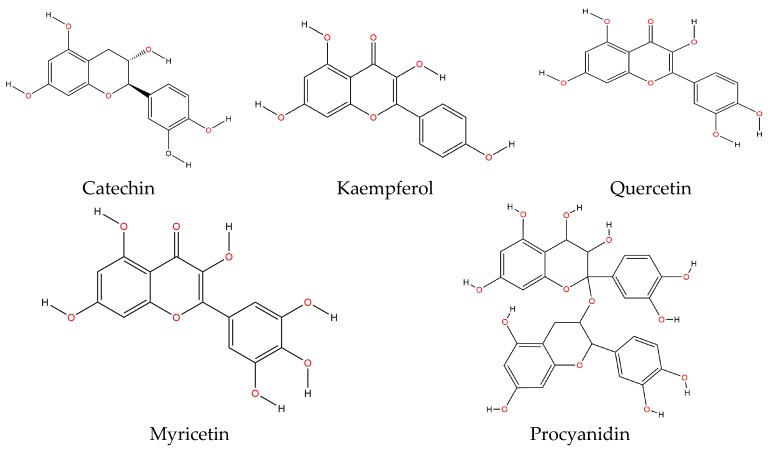
Structures of major flavonoids of *P. vulgaris* [46].

Flavonoids contained on the common bean are phenolic compounds that have been reported to act as inhibitors of tumor growth and some cancer types. These, along with phenolic acids and tannins, confer to this food a superior antioxidant capacity [9,49]. Flavonoids share a common structure consisting of two aromatic rings that are linked through three carbons, forming an oxygenated heterocycle. These are classified in six subclasses, depending on their heterocycle: flavonols, flavones, isoflavones, flavanones, anthocyanidins and flavanols (catechin and proanthocyanidin or condensed tannins). The main flavonoids contained in both raw and cooked bean are catechin, kaempferol, quercetin, myricetin and procyanidin [49] (Figure 2).

Flavonoids biological activity depends on the type of phytochemical constituents and the complexity of their structure and the composition of the flavonoids mixture, since it has been well established that phytochemical mixtures on fruits and legumes may provide protecting benefits to health, mainly through a synergic effect between them [56].

The consumption of these flavonoids has been inversely correlated with lung cancer and the risk of cardiovascular diseases (CVD). The putative mechanism of action involves the modulation of detoxifying enzymes and the inhibition of cell proliferation, although its most recognized effect is their antioxidant capacity [57]. It has been also found that flavonoids prevent platelet aggregation and induce muscle relaxation and, along with proteoglycans, they display an inhibitory effect of allergy symptoms. Additionally, flavonoids such as procyanidin B1 and resveratrol may enhance brain capacity and longevity [22]. Conversely, quercetin has showed a wide range of biologic activities, including anti-carcinogenic, anti-inflammatory, antiviral activities as well as decreased lipid peroxidation, platelet aggregation and capillary permeability. Moreover, this flavonoid displayed anti-inflammatory and immune properties in vitro (cells) and in vivo (animals). However, the studies conducted on humans did not completely support these results. The effect of quercetin as immune reinforcement on humans needs to be studied in detail before implementing a wide application in the future [58].

A number of epidemiological studies have also shown that diet flavonoids are linked to a low incidence of degenerative diseases such as CVD, type 2 diabetes, dementia and cancer. For example, flavonols showed a protective effect against type 2 diabetes in a cohort study [59], and anthocyanidins, flavan-3-ols, flavones, and flavonols were individually associated with a decreased mortality rate caused by CVD in the Cancer Prevention Study II Nutrition Cohort [60]. Nevertheless, a considerable knowledge gap still exists in this field. Other studies have reported inconsistent associations [61,62,63], but the underlying mechanisms have not been fully clarified [64]. Despite research which has shown that flavonoids have beneficial effects on health, there is no dietary reference intake (DRI) for this compound. In 1998, phenols, polyphenols, and flavonoids were excluded from the DRI panel’s consideration due to lack of food composition data and knowledge of actual intake amounts and limited information on their absorption and metabolism. The DRI committee report concluded that although these components “may be important dietary constituents, insufficient data are available at this time” [65]. Thus, many more studies are required along with long-term trials in order to establish diet recommendations for these compounds [65]. One of the main difficulties is the ability to safely estimate a safe consumption for these bioactive compounds. This, along with the elucidation of their main food source, is the first step in order to evidence a correlation between flavonoids and disease [66]. In this regard, some researchers made an effort to build and to analyze a database reporting the flavonoid content on several foods as well as the consumed amounts by some groups at different locations. For example, Jun et al. [66] estimated the overall and individual consumption of flavonoids in Korean adults and they identified the main sources of this compound. Thus, they found that the average consumption of total flavonoids per day in this particular group was 318 mg/day and they were comprised by proanthocyanidins (22.3%), flavonols (20.3%), isoflavones (18.1%), flavan-3-ols (16.2%), anthocyanidins (11.6%), flavanones (11.3%) and flavones (0.3%). Moreover, the food groups that contributed the most to this consumption were fruits (54.4%), vegetables (20.5%), legumes and their products (16.2%) as well as beverages and alcohol (3.1%). Furthermore, it was found that the main food contributing flavonoids on the diet were apples (21.9%), tangerines (12.5%), tofu (11.5%), onions (9.6%) and grapes (9.0%). In a similar study, Sebastián et al. [67] reported that the average daily consumption of total flavonoids by American adults was 251 mg/day; a lower value when compared to that consumed by Koreans, and flavan-3-ol represented the 81% of the intake. They found that the highest consumption of total flavonoids was observed for non-Hispanic white Americans (275 mg/day), followed by non-Hispanic Black (176 mg/day) and finally the Hispanic population (139 mg/day). The main source of these bioactive compounds was tea (80%). Peterson et al. [68] reported that the highest consumption of total flavonoids per d was in the United Kingdom (1017 mg/day), followed by Australia (775 mg/day) and they pointed out that the imprecision for estimating bioactive compound consumption, such as flavonoids, is challenging. The imprecisions may have originated from the variability of these compounds on food caused by the different culture and processing conditions, the quantification methods in the laboratory, incomplete food composition charts as well as the lack of a suitable instrument for diet evaluation.

Other studies were conducted to evaluate the effect of oral supplementation of quercetin in healthy individuals. For example, Egert et al. [69] investigated the effects of an oral supplementation of quercetin at three different doses on plasma concentrations of quercetin, parameters of oxidant/antioxidant status, inflammation, and metabolism. To this end, 35 healthy volunteers were randomly assigned to take 50, 100, or 150 mg/day (group Q50–Q150) quercetin for two weeks. Fasting blood samples were collected at the beginning and end of the supplementation period. Compared with baseline, quercetin supplementation significantly increased plasma concentrations of quercetin by 178% (Q50), 359% (Q100), and 570% (Q150). These authors reported that daily supplementation of healthy humans with graded concentrations of quercetin for two weeks dose-dependently increased plasma quercetin concentrations but did not affect antioxidant status, oxidized LDL, inflammation, or metabolism. However, to date there is no DRI for quercetin [65].

Table 3 shows the flavonoid profile for the Mexican black bean of the “Negro San Luis” variety. It exhibited a total flavonoid content of 765.50 mg/100 g of sample. In this variety, the most important flavonoids were quercetin 4-*O*-galactoside, myricetin 3-*O*-glucoside and kaempferol 3-*O*-glucoside [45]. Quercetin is also in skins of fruits, leafy vegetables, and berries, as well as in black tea, red wine, and various fruit juices [70]. Hertog et al. [71] reported quercetin content in onion of 284–486 mg/kg, in broccoli of 30 mg/kg, pear 6.4 mg/kg, and in different apple varieties of 21–72 mg/kg. These values are lower than those reported in raw black bean “Negro San Luis” variety [45] (Table 3).

Other studies also showed the presence of quercetin and kaempferol in several Mexican bean varieties, both wild-type and domesticated. In these samples, quercetin was within the following ranges: 6.9–23.5 μg/g of cooked bean and 4.3–12.0 μg/g of raw bean. On the other hand, kaempferol was within the ranges 13.8–209.4 μg/g of raw bean and 7.1–123.2 μg/g of cooked bean. The study observed that black bean displayed the highest quercetin content, whereas kaempferol was the highest in bayo beans [57]. A study conducted by Espinosa-Alonso et al. [14] on 63 Mexican wild-type bean lines reported both kaempferol and quercetin as being the main flavonoids. 

##### Condensed Tannins

The color of the bean seed coat is attributed to the presence and the amount of polyphenols such as flavonols glucosides, condensed tannins and anthocyanins. Their function is to provide protection against pathogens. These compounds display antioxidant, anti-mutagenic, anti-carcinogenic properties and also as free radical scavengers [14]. According to Guzmán-Maldonado et al. [72], the seed coat contains most of the tannins in beans, whereas their concentration is low in cotyledons.

Tannins are polymeric flavonoids that comprise a small part of the widely diverse group of phenolic compounds produced by vegetables as secondary metabolites. Along with oxalates and phytates, they are considered as anti-nutritionals because they affect nutrient bioavailability for the consumer. However, they are also considered as nutritionally important as they are antioxidants and potential anti-carcinogenic [64,73]. Condensed tannins or proanthocyanidins constitute a group of polymers and oligomers of polyhydroxy-flavon-3-ol linked through carbon-carbon bonds among the flavanol subunits. Their multiple phenolic hydroxyl groups allow their binding to proteins, metallic ions and other macromolecules as polysaccharides in order to form complexes [65,74]. They are considered bioactive compounds because their antioxidant, anti-carcinogenic and anti-mutagenic properties have been demonstrated [17]. Juárez-López and Aparicio-Fernández [13] reported a tannin content of 10.65 mg catechin equivalents (CE)/g on the “Flor de Junio” variety of the Mexican bean, a seed displaying pink spots on its seed coat. They also reported 2.15 mg CE/g on the “Peruano” variety of the Mexican bean that is characterized by a light yellow color. This suggested that the color on seed coat is directly correlated with the content of these compounds. These authors reported that thermal treatment affected the amount of condensed tannins on both studied varieties. This may be caused by the destruction of phenolic compounds or by changes in their structure or solubility. Iniestra-González et al. [16] reported a condensed tannin content of 10.5 and 10.56 mg CE/g in the Mexican bean varieties “Flor de Mayo Bajío” and “Flor de Mayo M38”, respectively. These share features with the “Flor de Junio” variety. González de Mejía et al. [17] reported higher values for these compounds on the Jalisco races of the common bean: “Flor de Mayo Criollo” (29 mg CE/g), “Flor de Mayo M-38” (38 mg CE/g) and “Flor de Junio Marcela” (32.9 mg CE/g), as well on the Durango races: “Bayo Victoria” (16.8 mg CE/g) and “Pinto Villa” (19.9 mg CE/g). They were cultured on five different sites within the semi-arid region in Mexico. Almanza-Aguilera et al. [12] reported that the condensed tannins content considerably decreased after the cooking (94%) and frying (95%) processes in 16 Mexican genotypes. This shows that seed maceration eliminates of most of these compounds. It is recommended to use the water resulting from this process for cooking purposes in order to preserve them on the final product. These authors found that bean cultivar “Negro Durango” possessed the highest content of condensed tannins after its cooking. The observed value was 132 mg CE/100 g, followed by “Negro Guanajuato” with 68.8 mg CE/100 g. After the frying process, “Negro 8025” contained the highest amount of this bioactive compound with a 74.9 mg CE/100 g value. A study carried out by Reynoso et al. [18] showed that the bean variety “Flor de Junio Marcela” possessed a high tannin content (698.4 mg CE/100 g) when compared to other cultivars of this grain. This content is 3.5-times higher regarding the “Pinto Zapata” bean (197.9 mg CE/100 g), nine-times higher when compared to the “Flor de Mayo Anita” variety (75.6 mg CE/100 g) and over thousand-times regarding “Blanco Tlaxcala” (0.60 mg CE/100 g). Guzman-Maldonado et al. [63,74] reported tannin content within the 6.9–32.4 mg CE/g range in 19 common bean varieties grown in the states of Aguascalientes and Durango (Mexico). These authors mention that there is an undetected amount of condensed tannins; consequently, the actual content of these compounds is underestimated. In their study, they found 9.9–66.4% of undetected tannins in the Aguascalientes varieties and 4.1–69.7% in those from Durango. Another study conducted by Espinosa-Alonso et al. [14] on 62 Mexican wild-type bean lines reported that the amount of condensed tannins is 9.49–35.70 mg CE/g of flour. This study also analyzed the content of condensed tannins in the “Negro Jamapa” and “Frijol Pinto” varieties and the following values were found: 30.86 and 21.37 mg CE/g of flour, respectively.

##### Anthocyanins

Anthocyanins are classified as phenolic compounds, particularly as flavonoids. They provide pigments to vegetables and they are widely distributed in nature. They confer color to the bean seed coat on the red, black and pink-colored varieties. Juárez-López and Aparicio-Fernández [13] assessed the amount of anthocyanins on the Mexican variety “Flor de Junio” and they found 0.43 mg equivalents to cyaniding 3-glucoside (EC3G)/g of raw bean. They reported a significant effect caused by processing: a decrease correlated with the intensity of the thermal treatment. Other studies have found three types of anthocyanins in the black bean: delphinidin 3-glucoside (56%), petunidin 3-glucoside (26%) and malvidin 3-glucoside (18%) [66,75]. Salinas-Moreno et al. [67,76] also reported the former anthocyanidins as predominant in 15 Mexican varieties of the Jalisco, Mesoamerica and Recombined races. This suggests that these compounds possess a high antioxidant activity and they prevent diseases such as cancer, atherosclerosis and inflammation. Conversely, Díaz et al. [75] reported low anthocyanin content in the Mexican genotypes of the yellow-colored common bean or their combinations when compared to the high content on the red-colored beans. This observation indicates that the level of these bioactive compounds is higher in dark-colored beans in comparison to those light-colored. Reynoso et al. [18] reported an anthocyanin content of 3.75 mg EC3G/100 g in bean cultivar “Flor de Junio Marcela”. This content was higher when compared to that found on other cultivars such as “Pinto Zapata” (1.77 mg EC3G/100 g), “Flor de Mayo Anita” (0.0 mg EC3G/100 g) and “Blanco Tlaxcala” (0.09 mg EC3G/100 g). Espinosa-Alonso et al. [14] studied 62 Mexican wild-type bean lines and they identified delphinidin, petunidin, cyanidin, malvidin, pelargonidin and peonidin as the main anthocyanins on the analyzed samples. They reported total anthocyanidin content within the 0.01–1.85 mg EC3G/g range in all of the studied varieties. Martínez et al. [15] evaluated the anthocyanin content on the “Negro Jamapa” and “Mayocoba” varieties of the Mexican bean and they only found the previously mentioned compounds on the former. The main anthocyanins were delfinidin (47%), petunidin (22%) and malvidin (13.53%). Additionally, they measured a higher content of total pigments in the “Negro Jamapa” peels (13.53%) regarding “Mayocoba” (6.7%). They also observed that anthocyanidins, similarly to flavonoids, were degraded by thermal treatments at high temperatures and/or prolonged thermic contact. After a pre-cooking process the levels contained on its raw form decreased from 20% up to 80% after the cooking and canning processes. This effect was pronounced on the cultivar “Negro Jamapa”; thus, it was identified as the most sensitive towards thermal treatments.

Other sources of anthocyanins are strawberry (7–66 mg/100 g), cherry (3–230 mg/100 g), blackberry (28–366 mg/100 g), and red raspberry (28–116 mg/100 g) [77]. These results are higher than those obtained in Mexican bean cultivar “Flor de Junio Marcela” [18].

Although anthocyanins offer great health benefits, dietary reference intakes do not currently exist for anthocyanins and many other dietary bioactive compounds in the United States, Canada, or the European Union. China has currently defined a specific proposed level of 50 mg/day for anthocyanins, while the Joint FAO/WHO Expert Committee on Food Additives has established an acceptable daily intake of 2.5 mg/kg per d for anthocyanins from grape-skin extracts but not for anthocyanins in general. After a request from the European Commission to the European Food Safety Authority, the Scientific Panel on Food Additives and Nutrient Sources Added to Food was asked to provide a scientific opinion re-evaluating the safety of anthocyanins. The panel concluded that the currently available toxicologic database was inadequate to establish a numerically acceptable daily intake for anthocyanins [78].

##### Isoflavonoids

Isoflavonoids are a subgroup of flavonoids, are phytoestrogens that exhibit pseudohormonal properties as a result of their functional and structural similarity to the natural estrogen 17β-estradiol and may interact with estrogen receptors. Isoflavone content is variable among plant species and may even vary among genotypes of the same species. In addition, isoflavone content may also be affected by external factors related to crop location, such as temperature, fertilization levels, occurrence of pests and diseases, time since harvest, farming practices, and processing and food preparation methods [79].

The primary isoflavones in soybeans are genistein (4′,5,7-trihydroxyisoflavone) and daidzein (4′,7-dihydroxyisoflavone) and their respective b-glycosides, genistin and daidzin (sugars are attached at the 7 position of the A ring) [80].

Common bean is not recognized as an isoflavone source, the soybean is the legume that exhibits the highest content of these compounds [57]. However, some studies have shown the presence of these compounds in common bean. Lima et al. [79] reported the presence of nonglycosylated forms of isoflavonoids daidzein and genistein in 16 genotypes of Brazilian common bean germplasm. These authors found that grains of the black type showed the highest concentrations of isoflavonoids and were the only ones to exhibit daidzein. However, the isoflavonoid content obtained in the bean genotypes evaluated was very low compared to that obtained in soybean grains.

Diaz-Batalla et al. [57] analyzed ten cultivated and four wild varieties of Mexican common bean seeds, and reported that the presence of daidzein and genistein in raw and cooked bean seed flour was not confirmed by the UV spectra. However, during the germination of the seven common beans seed samples (five cultivated and two wild), there was daidzein and genistein genesis, and the values for daidzein ranged from 8.2 to 129.1 μg/g, those for genistein from 2.6 to 9.7 μg/g. Similar results were obtained by Guajardo-Flores et al. [45], in Mexican black bean “Negro San Luis” variety, these authors reported that Genistein was detected only after three d of germination, in concentrations that ranged from 0.12 to 0.31 mg/100 g. Higher results were reported for soybean (600 μg/g and 950 μg/g respectively) [81]. 

Daidzein, exhibits anticancer effects; e.g., it inhibited the growth of HL-60 cells implanted in the subrenal capsules of mice. However, genistein has attracted most of the interest. There are literally hundreds of in vitro studies showing that genistein inhibits the growth of a wide range of both hormone-dependent and hormone independent cancer cells with an IC50 between <5 and 40 mM (2–10 mg/mL), including breast, prostate, colon, and skin cells. Also, in vitro, genistein inhibits the metastatic activity of both breast and prostate cancer cells independent of the effects on cell growth [2]. The effect of isoflavones is not exclusively hormonal; genistein is a specific inhibitor of protein tyrosine kinases and DNA topoisomerases I and II and arrests cell grow by interfering wth the signal transduction pathways. Additionally, phytoestrogens exhibit antioxidant activity [57]. 

#### 4.2.2. Phenolic Acids

**Figure 3:**
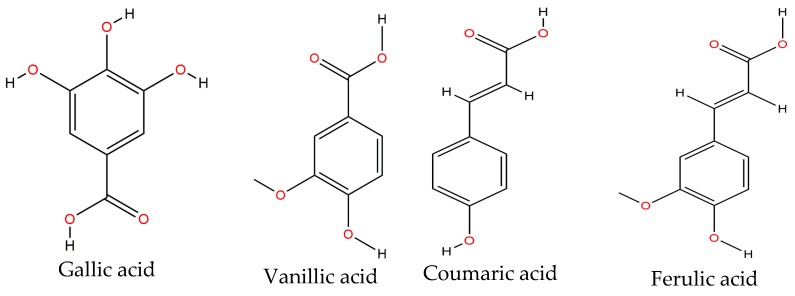

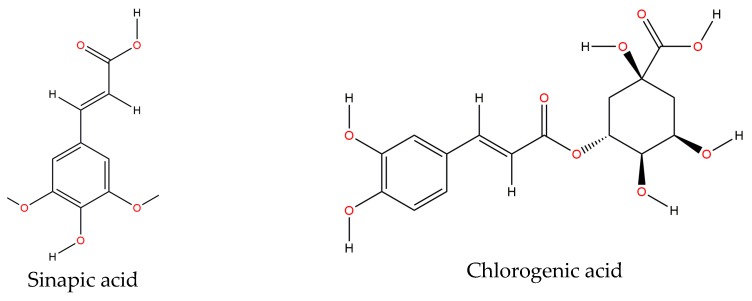
Structures of major phenolic acids of *P. vulgaris* [46].

Phenolic acids are of great importance in vegetables as they are precursors of other more complex phenolic compounds. These may be classified in two types: those derived from benzoic acid (e.g., *p*-hydroxybenzoic, vanillic and gallic acids) and those derived from cinnamic acid (e.g., ferulic, p-coumaric, and caffeic acids) [57]. Gallic, vanillic, coumaric, sinapic, ferulic and chlorogenic acids (Figure 3) are mainly found on the common bean, either raw or cooked. A study conducted by Espinosa-Alonso et al. [14] showed that ferulic acid is the main phenolic acid on 62 lines of the wild-type Mexican bean.

Some studies have shown that cooking does not affect the content of these phenolic acids [49], although some authors have reported otherwise [82]. The effect of a thermal treatment has been also reported for some varieties of the common Mexican bean. Díaz-Batalla et al. [57] reported decreased levels of p-hydroxybenzoic acid in the raw form when compared to the cooked form. The quantified values were 5.7–13.8 μg/g and 4.5–8.6 μg/g, respectively. The decrease after processing changed from 17.9 to 44.5%. Furthermore, vanillic acid changed from 5.2–16.6 μg/g in raw bean to 3.5–12.1 μg/g in its cooked form. Thus, the decrease caused by cooking was 12.5 to 36.9%. Coumaric acid was also reported on these samples within the 3.2–6.8 μg/g and 1.7–4.7 μg/g ranges for the raw and cooked forms, respectively, that represented a 26.3–66.3% decrease. Ferulic acid changed from 17.0–36.0 μg/g in raw bean to 11.9–27.9 μg/g in cooked bean, implying a decrease from 15.5% to 36.5%. Martínez et al. [15] also quantified gallic, p-hydroxybenzoic, vanillic, ferulic and coumaric acids on the “Jamapa” and “Mayocoba” varieties of the black bean in their raw and cooked forms. They reported that thermal processing only affected gallic and p-hydroxybenzoic acids, whereas the rest remained constant.

Regarding their biologic activity, it has been found that phenolic acids display anthelmintic activity, they prevent sickle red cells and suppress hepatic fibrosis in chronic liver disease [49]. Some studies have reported that gallic acid possesses bacteriostatic properties, it counteracts melanoma onset and it functions as an antioxidant and it has been also proposed for the treatment of brain tumors. On the other hand, chlorogenic acid protects against neurotoxins by decreasing apoptosis induced by the beta-amyloid peptide. It also displays of anticholinesterase, radical scavenger and anti-amnesic activities. Ferulic acid also displays beneficial effects on health as antioxidant, anti-inflammatory and inmunostimulat. It also promotes the degradation of the recombinant beta-amyloid peptide. Caffeic acid also displays a neuroprotective effect against beta-induced toxicity as it inhibits calcium efflux and Tau phosphorylation. It also protects neurons against oxidative stress-induced cytotoxicity and, along with coumaric acid, it may induce neuroprotective effects against Parkinson disease in a similar manner as previously observed with flavonoids, catechin and quercetin [83].

### 4.3. Polysaccharides

#### 4.3.1. Dietary Fiber

The American Association of Cereal Chemists (AACC) defines dietary fiber as “the edible part of plants or analogue carbohydrates that are resistant towards digestion and absorption on the small intestine that are wholly or partially fermented on the large intestine”. This classification includes polysaccharides, oligosaccharide, lignin and substance related to vegetables [84]. Dietary fiber plays a crucial role in food structure, carbohydrate availability, starch cleavage and consequently for the glycemic index of foods. Therefore, the management of corporal weight and the structure of the diet, including diabetes prevention and metabolic syndrome, may be linked to dietary fiberconsumption [85]. Some studies have demonstrated a benefit from dietary fiber consumption for the treatment of type 2 diabetes mellitus. Fujii et al. [86] reported that a high consumption of this compound correlated with improvements of glycemic index, including risk factors for cardiovascular diseases such as abdominal obesity, hypertension and metabolic syndrome. This was also the case for chronic kidney disease in Japanese patients with type 2 diabetes. Fiber was obtained from vegetables, cereals, legumes and bean.

Huang et al. [87] conducted an observational study and they built a database comprising 25 eligible studies and 42 cohorts that included 1,752,848 subjects with an average study follow-up period of 12.4 years. When compared to individuals with low fiber intake, the mortality rate caused by cardiovascular diseases decreased by 23%, the one due to cancer decreased by 17%, digestive diseases by 68%, infectious diseases by 58%, and inflammatory diseases by 43%, in those individuals consuming high amounts of fiber. Furthermore, these authors reported that for each 10 g of total consumed fiber the mortality risk associated to coronary disease could be decreased by 27%.

Several mechanisms have been suggested in order to explain the protective effects on health derived from fiber intake and its components. The decreased risks of coronary disease, hypertension, cerebrovascular accidents, diabetes and obesity are the consequence of an improvement of serum lipid levels, immune function, and laxation. The latter increases the stool volume as well as the transit time along the intestine allowing slow glucose absorption and improving sensibility towards insulin. Additionally, some diet fibers are fermentable and their catabolisms on the intestinal tract generate several bioactive compounds that may significantly increase biomass and modify the flora composition [87].

Dietary fiber may be classified in soluble and non-soluble fractions, according to their solubility during its extraction and isolation in an enzyme solution with controlled pH (representing the human food enzyme) [88].

Soluble dietary fiber includes oligosaccharides, fructo-oligosaccharides, pectins, β-glycans, galactomannan gums, alginate and psyllium, whereas its non-soluble counterpart contains mainly cellulose, hemicellulose, resistant starch and lignin [85].

Soluble and non-soluble fiber diets possess a number of beneficial properties for health, weight loss, satiety increase, effects on inflammatory markers and on intestinal microbiota. Many of such benefits are derived from the viscous nature caused by soluble fiber consumption, including the prevention of macronutrient absorption, the slowdown of gastric emptying, and decreased postprandial responses towards glucose, hypocholesterolemic effects and fermentation at the colon. Furthermore, an increased sensibility towards insulin is one of the factors contributing to the beneficial effects of non-soluble fiber. Additionally, it allows water absorption and it has intestinal regulatory effects. These physiologic differences mainly depend on the structure and physical properties of each fiber type [85]. Moreno-Franco et al. [3] found an inverse correlation between non-soluble fiber consumption and systolic and diastolic blood pressure, overall cholesterol, triglycerides, apolipoproteins B100 and the triglycerides (TG)/high-density lipoprotein (HDL) ratio. A higher control of hypertension, metabolic syndrome, and lipid profile management was achieved by an increased intake of non-soluble fiber.

The common bean contains carbohydrates that are slowly digested and a high proportion of non-digestible carbohydrates that are eventually fermented on the large intestine. Half a cup of beans provides 5.2–7.8 g of total fiber, that account for 20–31% of the daily recommended consumption values for adults in several parts of the world (25–35 g); half a cup of beans also provides 0.6–2.4 g of soluble fiber [2,38].

A consumption of 2–10 g/day of soluble fiber has been correlated with a small but significant decrease of total cholesterol [89]. Other authors report that 5–10 g of soluble fiber per d reduces LDL cholesterol approximately by 5% [2]. Therefore, the importance of encouraging an increased consumption of common bean in the diet.

Table 4 presents the soluble and non-soluble fiber content on the Mexican bean varieties, either cooked or raw, as reported by Campos-Vega et al. [38]. These authors found a higher content of both soluble and non-soluble fiber in cooked beans. The “Bayo Madero” bean exhibited the highest concentration of both fractions when compared to the other studied varieties. 

#### 4.3.2. Oligosaccharides

These are important bioactive compounds contained in beans. They are considered as prebiotics because they are hydrolyzed at the colon and they are fermented by intestinal bacteria thus producing short-chain fatty acids such as acetic, propionic and butyric. These are correlated with prevention against colon cancer caused by azoxymethane. However, they also produce carbon dioxide, hydrogen and methane, the gasses linked to flatulence caused by the inability in humans to cleave the oligosaccharides raffinose, stachyose and verbascose due to the absence of the enzyme α-galactosidase [90,91]. Short-chain fatty acids in the colon are important cellular nutrients. The levels of these acids may also stimulate the absorption of water and consumed minerals resulting in a fast recovery from malaises such as diarrheas and to prevent mineral deficiencies. Butyrate, a four-carbon fatty acid, is physiologically relevant for colon epithelium as it promotes the proliferation of normal mucosa. The interest on this fatty acid as protective agent derives from its diverse physiological properties as well as its anti-proliferative effects on transformed cells. Moreover, it has been reported that this acid induces apoptosis, inhibits proliferation and leads to a highly differentiated phenotype in human colon carcinoma cell lines. Additionally, the propionic acid metabolism carried out in liver may suppress cholesterol synthesis and it may enhance butyrate anti-proliferative properties whereas acetic acid is absorbed, transferred to the bloodstream and metabolized in muscle, kidney, heart and brain tissues [38].

Iniestra-González et al. [16] reported the oligosaccharide content of 16 varieties of the Mexican common bean and it is presented in Table 5. This study observed higher amount of these bioactive compounds on the “Flor de Mayo M38” bean with 57.6 mg/g. The lowest value was observed in the “Flor de Mayo Bajío" bean with 39 mg/g.

Herrera et al. [55] reported that location, humidity regime and genotype impacted on the oligosaccharide content in eight genotypes of the common bean belonging to three Mexican races (Jalisco, Durango and Nueva Granada). Although, they did not find an effect on these compounds derived from cooking. These authors measured a lower oligosaccharide content on the raw seed of the Jalisco race varieties when compared to the Durango and Nueva Granada races. These authors also found that “Azufrado Noroeste” beans from the latter race exhibited the highest oligosaccharide content thus explaining the sweet taste of yellow-colored beans.

Díaz-Batalla et al. [57] reported the raffinose, stachiose and verbascose content on some genotypes of the Mexican common bean. In this study, they found a raffinose content of 4.4–11.4 mg/g and 3.2–11.4 mg/g on raw and cooked seeds, respectively, a processing-induced decrease of 1.7–36.8%. Stachiose was within the 50.9–63.8 mg/g and 37.9–56.7 mg/g ranges on the raw and cooked forms, respectively, a decrease of 3.8–25.5%. Finally, verbascose was 2.2–5.1 mg/g and 1.6–4.4 mg/g on raw and cooked seeds, respectively, representing a 1.7–43.3% decrease. Campos-Vega et al. [38] reported stachinose, raffinose and verbascose as the main oligosaccharides on both raw and cooked beans of the “Negro 8025”, “Bayo Madero”, “Pinto Durango” and “Azufrado Higuera” Mexican varieties. Among their results, these authors observed an increased stachinose concentration after cooking in all analyzed samples. Similarly, an increased verbascose content was observed on some varieties such as “Negro 8025” and “Azufrado Higuera”. The values were lower when compared to those obtained by Díaz-Batalla et al. [57] with other Mexican genotypes. In the study conducted by Campos-Vega et al. [38] they reported that the carbohydrates obtained from the common bean varieties were fermented in vitro and they modified the colon’s pH through the production of short-chain fatty acids. The highest yield of the latter was observed on the “Negro 8025” and “Bayo Madero” varieties thus conferring great benefits to this section of the stomach.

#### 4.3.3. Resistant Starch

According to its nutritional features, starch is classified as digestible or resistant. The former is rapidly digested on the small intestine because of the gelatinization suffered by starch grains during cooking and their susceptibility for enzymatic attack, whereas the resistant form comprises starch and its degradation products that are not absorbed by the small intestine of healthy individuals. Thus, these are absorbed by the large intestine and their degradation produces lactic, succinic and propionic acids. These promote the growth of intestinal flora. Resistant starch is highly valued by the food, pharmaceutical and chemical industries [92]. The types of resistant starch on foods are: the starch trapped within the whole or partially ground grain, the native form, the type B non-gelatinized starch granules and retrograde starch [93].

Legumes contain significant amounts of resistant starch when compared to other products such as cereals, tubers and immature fruits. This content may explain the low digestive rate and the glucose release to the bloodstream after vegetable consumption, resulting in a decreased glycemic, insulinemic and postprandial responses compared to cereals or potatoes. Resistant starch is becoming a subject of interest as it has highly beneficial effects on health such as the modification of the colon environment through the production of short-chain fatty acids, mainly butyric acid, whereas it reduces the amount of the potentially toxic products derived from protein fermentation. Resistant starch also protects against tumorigenesis and it decreases the effects caused by tumor abundance caused by non-digestible proteins from foods [38].

Beans contain high amounts of resistant starch. Its content its higher when compared to that of other commonly consumed grains, mainly because of its high amylose/amylopectin ratio. This bioactive compound, along with diet fiber, confers the bean with the ability to decrease the glycemic index in comparison with other carbohydrate-rich foods, thus producing great benefits on health. The glycemic index for bean is within the 29–38 range, whereas for integral rice is 50 and 55 for oat flakes [2].

Campos-Vega et al. [38] reported the resistant starch content on the Mexican varieties of the common bean. It was 37% on “Bayo Madero”, 32% on “Negro 8025”, 28% on “Pinto Durango” and 34% on “Azufrado Higuera”. These concentrations were higher when compared to those obtained by Osorio-Díaz et al. [94] on other Mexican varieties such as “Negro” and “Flor de Mayo” beans. These authors found that resistant starch was higher on bean flour in comparison to whole grain and no significant effect was observed after storage. Kasote et al. [95] mentioned that processing such as maceration and pressure cooking are useful in order to increase the amount of resistant starch in several foods, including beans. However, in Mexico the most common cooking method for this food is at atmospheric pressure without previous maceration because this processing does not affect its taste. Physicochemical changes of the grain occur during cooking, such as protein denaturalization, polysaccharide solubilizing, softening and the disruption of the seed’s middle lamellae and starch gelatinization. These changes improve texture, the nutritional value and bean starch digestibility that is relatively low when compared to other starch sources. This decreased digestibility is due to intrinsic factors such as crystalline structure and the structural features of amylopectin, but also to extrinsic factors such as diet fiber that promotes increased viscosity on the small intestine, thus decreasing the accessibility of the starch enzyme [39].

Bean starch digestibility and physical chemical properties may be affected by cooking, the hydric regime of the culture and the specific variety. Ovando-Martínez et al. [39] reported that resistant starch from the “Negro 8025” and “Pinto Durango” beans decreased with traditional cooking but no differences were observed when comparing hydric regimes and varieties. However, an increased content was observed in beans cultured under irrigation when compared to those obtained with weather conditions (fed by the rain). This starch fraction was within the 12.20–30.43% range on the raw bean, whereas it was within 5.80–8.14% on its cooked form. Resistant starch digestibility was also higher in cooked samples regarding those in their raw form and “the Negro 8025” variety displayed the lowest glycemic index and digestibility values when compared to the cooked “Pinto Durango” variety.

### 4.4. Lectins or Agglutinins

Lectins are a diverse group of proteins with great affinity for carbohydrates and biomolecules that contain carbohydrates, including a variety of sugars, modified sugars, proteins and lipids. This complementarity is highly specific and allows lectins to have an effect on a wide range of biologic processes [96]. Other authors state that lectins are glycoproteins of non-immune origin that possess a high affinity towards the carbohydrate moieties on glucoconjugates [97] occurring on the cell’s surface [82]. They may be derived from plants, microorganisms or some animals and they may be either soluble or be bound to the plasma membrane. Generally, they are classified as four groups according to their affinity with glucose/mannose, galactose, *N*-acetyl-d-galactosamine, l-fructose and sialic acid. Another classification is based on the type of protein: type 1, depending on structural sequence and evolutionary similarities, or type 2 depending on proteins but not evolution. Some examples of structurally differentiated lectins are erythrin C, concavalin C, Ulex europaeus and type C [98].

The term lectin was proposed by Boy and Shapleigh in 1954 from the Latin “Legere” (selected, chosen) and it refers to their ability of selective binding to particular sugars. This term was later generalized in order to encompass all sugar-binding proteins and those inducing cell agglutination of non-immune origin as found on animals, vegetables and microorganisms [97,99]. Lectins of vegetable origin possess a great variety of biologic activities including cell agglutination, mitosis, toxicity and inhibition of cell growth. Several types of lectin have shown to induce cell death of cancer cells, suggesting that they may have an application for cancer treatment. Carcinogenic cells secrete or express glucoconjugates with abnormal glycan structure and lectins may detect such changes. Based on this property, they may be used for cancer diagnosis or in some specific treatments for this disease [97]. An anti-HIV activity has also been reported for some lectins [100]. They may also potentially be used as nutraceuticals to control obesity. This may be attributed to their ability to resist gastric digestion and to be absorbed by the bloodstream while remaining biologically active. These properties have resulted in increased research in order to use them for cancer prevention purposes [101].

Lectins of vegetable origin are commonly found in foods consumed without processing, such as fruits and vegetables. Green peas, chickpeas, lentils and other legume seeds contain the highest amount of lectins among all groups. It is thought that these compounds act as the plant protective defense mechanism against predators. This feature has allowed their use as bioactive insecticides. However, when legumes are consumed in their raw form they may be resistant against digestion causing adverse effects on the consumer [101].

Lectins are present on the common bean, as well as protease inhibitors, trypsin inhibitors, some polyphenols and phytic acid. All of the latter are considered as anti-nutrient. However, it is thought that these compounds do not have a negative effect on human health as their activity decreases with cooking or processing [2,57,102]. Some evidence suggests that these compounds provide beneficial effects to human health because of their role in disease prevention. Therefore, they are defined as nutraceuticals [17].

It has been reported that lectins contained in the common bean impair the growth of non-Hodgkin lymphomas, a lymphoid tissue cancer that invades lymphatic ganglia, the spleen and other immune organs. They may be also used as tumor markers because they identify those cells undergoing the first differentiation stages towards cancer cells [5,35]. Pervin et al. [98] reported the inhibition of cell proliferation of the MCF7 breast cancer cell line, the HepG2 hepatoma cell line and the nose-pharyngeal carcinoma CNE1 and CNE2, induced by the presence of these compounds contained on the common bean.

Lectins from several sources have been also identified as active immunomodulating agents that may enhance the immune system, lymphocyte proliferation, the natural-occurring cell death, antibody synthesis and cytokine regulation [101]. The current knowledge regarding the immunomodulating effects of lectins contained in legumes is based on the studies conducted on those extracted from the green pea (*Pisum sativum*) [82,103]. These lectins activated spleen lymphocytes in mice, but more studies are needed in order to characterize their effects on humans. Lectins obtained from legumes such as bean may be effective to treat obesity. It has been demonstrated that they decreased fat accumulation in rats. This effect was attributed to decreased insulin levels caused by the activity of these compounds. Therefore, they may function as therapeutic agents in human trials to stimulate grastrointestinal function and to decrease the incidence of obesity. It seems that the most commonly used lectins for this purpose are those contained in beans [101].

Some studies, such as the one conducted by González de Mejía et al. [17] have reported lectin activity in five varieties of the Mexican common bean (“Flor de Mayo criollo”, “Flor de Mayo M-38”, “Flor de Junio Marcela”, “Bayo Victoria” and “Pinto Villa”) that were cultured in five locations in high and semiarid lands in Mexico. The study discovered significant differences among zones and varieties. The average activity values were within a 1.5–2.3 hemagglutinating unit (HAU)/g range. The highest value was found for the “Bayo Victoria” bean, whereas the lowest was observed for “Pinto Villa”. It is known that lectins may be heat-sensitive. Based on this, some technologic processes have been proposed in order to inactivate them, such as traditional bean cooking. Paredes-López et al. [104] studied the effect of cooking and maceration on lectin inactivation on “Flor de Mayo” variety of the Mexican common bean, a cultivar with no resistance towards the mosaic virus, and on the genetically enhanced “Flor de Mayo” genotype with enhanced resistance towards the latter pathogen. The results showed that maceration decreased lectin activity on raw bean. However, no differences were observed when lectin activity was compared between the macerated and cooked beans and those without treatment. Additionally, the enhanced variety displayed higher activity when compared to wild-type. Traditional cooking virtually eliminated the activity on both materials. A biphasic pattern of thermal inactivation was identified regarding lectin activity. Coffey et al. [105] reported that macerating and cooking beans at high pH values was an effective way to reduce bean lectin activity and to decrease the time needed to reach a desirable texture.

### 4.5. Protease Inhibitors

They are defined as thermo-sensitive compounds of protein nature that modify protein digestion by inhibiting the activity of digestive enzymes dedicated to hydrolyze those proteins consumed on the diet. Those inhibiting serine-proteases are the most known, such as trypsin and chymotrypsin [44].

Protease inhibitors have been isolated from a wide variety of fruits, vegetables and legumes. Trypsin and chymotrypsin are the major protease inhibitors contained in legumes. It has been pointed out that if these are derived from green peas, chickpeas or lentils, they belong to the Bowman-Birk Inhibitors (BBI) family. The latter possess two active sites that inhibit these proteolytic enzymes. However, if they are derived from soy, they may belong to either BBI or to protease inhibitors of the Kunitz family [101].

Protease inhibitors are considered as anti-nutritional as they interfere with legume digestion or that of their flours if they are consumed in their raw form. These inhibitors are resistant to pepsin and to the environmental acid pH of the human digestive tract. Thus, they interfere with digestion by inhibiting trypsin and chymotrypsin via irreversible binding [101]. They act as inhibitors by suppressing the negative feedback regulation of pancreatic secretions through the release of the cholecystokinin hormone from intestinal mucosa. This may stimulate a pancreatic hypertrophy and decrease the host’s growth and performance because of the impaired ability of its enzymes to hydrolyze diet proteins. Consequently, this impinges on amino acid absorption and on de novo protein synthesis [106].

In spite of the fact that protease inhibitors are considered anti-nutritionals, several reports have mentioned the great potential they possess to promote human health. Additionally, it has been highlighted their use as anti-inflammatory agents to treat diseases such as HIV, hypertension, neurodegenerative diseases and several infectious conditions. However, these diseases are often treated with synthetic peptide-mimicking inhibitors instead of protease inhibitors obtained from natural legumes. The therapeutic use of the latter has been restricted only to some types of cancer or as anti-inflammatory agents [101]. It has been reported that trypsin inhibitors provide protection against rotavirus, they inhibit carcinogenesis and they may be used as chemoprotective agents in spite of their toxic role. No toxic effect has been reported during clinical or laboratory studies, even when they were dosed at high levels [5,35].

According to Wang et al. [107], trypsin inhibitors are low molecular weight proteins capable of inactivate digestive enzymes such as trypsin. However, the activity of these compounds significantly decreases with cooking. These authors reported an 87% decrease on cooked beans and chickpeas. Furthermore, Messina et al. [2] observed that by boiling beans the trypsin inhibitor content decreased by 80–90%. Roy et al. [101] showed that by combining maceration with thermal treatment the levels of protease inhibitors are decreased in green pea, chickpea and soy. Additionally, some agronomic practices such as the two-phase harvest elicited a 44% decrease of trypsin inhibitors in green peas and 25% in yam, although this decrease was not significant for lentil and soy. On the other hand, the genetic enhancement programs have tried to lower the levels of this compound in legumes through genetic manipulation. Nevertheless, it is possible that cultures do not respond in a proper manner because they may result in lower yields as protease inhibitors also protect the plant against predators and the seed against fungi and microorganisms during storage. 

The common bean is a source of trypsin inhibitors. González de Mejía et al. [17] reported an average content within the 7.9–11.9 trypsin inhibitor units (TIU)/mg range in the Mexican bean varieties “Flor de Mayo Criollo”, “Flor de Mayo M 38”, “Flor de Junio Marcela”, “Bayo Victoria” and “Pinto Villa”. The lowest value was observed for the latter, whereas the highest was for both “Flor de Mayo M38” and “Flor de Junio Marcela”. These authors suggested that cultivars from the Jalisco race are characterized by higher trypsin inhibitors content when compared to the Durango race. Such results were lower in comparison to those obtained by Guzmán-Maldonado et al. [74] on 17 varieties of the Mexican common bean as they reported a 12.9–23.4 (TIU/mg) range. It has been observed that those varieties possessing a more colored seed coat contain higher levels of trypsin inhibitors when compared to those characterized by lighter color, such as “Bayo Victoria” or “Pinto Villa”. Iniestra-González et al. [16] measured a trypsin inhibitor content of 4.78–12.22 (TIU/mg) on 16 varieties of the Mexican common bean. The “Flor de Mayo M38” variety contained the higher average levels, followed by “Negro Sahuatoba”. Conversely, that with the lowest content was the “Azufrado Peruano87” variety. These authors pointed out that the black- and pink-colored varieties contain the highest values of trypsin inhibitors, followed by those with golden or brown (Bayo) coloration, and finally those with a white or yellow seed coat (Azufrado).

### 4.6. Phytic Acid

Phytic acid is found on cereals, nuts, oilseeds and legumes in amounts from 1 to 5% of the seed’s weight. They function as phosphorous storage. It has been recognized as anti-nutrient because of its lack of digestibility and its ability to decrease bioavailability of protein- or starch-bound divalent and trivalent cations such as iron and zinc thus reducing their digestibility. Therefore, a diet with a high-energy content obtained from legumes and grains may lead to nutritional deficits. However, their interaction with starch and divalent cations cause a low glycemic index and it reduces iron participation in metal oxidations thus mediating the effects correlated with a low incidence of diabetes, cardiovascular diseases and the risk of colon cancer [57]. It has been also reported that phytic acid attenuates the risk of cancer, mainly breast and colon cancer, an effect probably due to its antioxidant capacity [5,35]. Phytic acid may inhibit the absorption of the iron obtained from foods as cereal grains and legumes because of its capacity to form non-absorbable complexes with iron on the gastrointestinal tract. This property has an essential biologic function in plants as it provides phosphorous and essential mineral to growing seedlings and it seems to participate in signaling and response toward pathogens. Therefore, the genetic improvement as a practice to decrease their levels and to increase the nutritional value of plants may have a negative effect on seeds and on the culture’s yield. Nevertheless, the decrease of phytic acid by genetic means through disruption of its biosynthesis pathway thus resulting on the selection of the lowest phenotype is being considered as a possible solution in order to attenuate the iron bioavailability issues in these foods [108].

The studies conducted by Iniestra-González et al. [16] on 16 enhanced varieties of the Mexican common bean showed a phytic acid content within the 0.98–2.16 mg/g range. The variety that contained the highest levels was “Mayocoba” whereas “Bayo Durango” exhibited the lowest. Díaz-Batalla et al. [57] reported the phytic acid content of cultured and wild-type varieties of the Mexican common bean within the 7.8–17.6 mg/g and 5.7–15.3 mg/g on their raw and cooked forms, respectively. These authors highlighted the fact that phytic acid status as anti-nutrient or bioactive compound does not depend on the phytic acid *per se* or its source. It rather depends on the diet’s diversity. On diets based on grains or legumes with low content of animal proteins, phytic acid compromises mineral balance and health, especially in susceptible individuals as children. As long as these subjects consume a diverse diet, in which micronutrient consumption and bioavailability are high, they may be positively affected by consuming this compound.

## 5. Antioxidant Capacity of the Common Bean 

The common bean has an antioxidant capacity because of the presence of phenolic acids, flavonoids and tannins. This capacity is mainly due to the reducing capacity of polyphenols as they play a very important role during free radical neutralization or scavenging as well as chelation of transition metals, thus impairing both initiation and propagation of oxidative processes. The intermediates formed as consequence of phenolic antioxidants activity are relatively stable because of the resonance within the aromatic rings contained in their structures [49].

According to Akillioglu and Karakaya [50], the antioxidant activity of the common bean is increased after digestion. This may be caused by the higher solubility of polyphenols as well as protein and starch digestion. This may favor the release of phenolic compounds due to the acid environment prevailing on stomach and to enzyme-mediated hydrolysis on the duodenum.

Some authors have reported an antioxidant activity on the varieties of the Mexican common bean and they have pointed out some of the factors that contribute to this variable such as thermal processing and variety. Juárez-López and Aparicio-Fernández [13] studied the varieties of the Mexican common bean “Flor de Junio” (characterized by pink spots on the seed coat) and “Peruano” (light yellow-colored). They found a higher antioxidant capacity on “Flor de Junio” (29%). This value is similar to that obtained with 300 μmol of the commercial antioxidant butylated hydroxytoluene (BHT), whereas “Peruano” bean displayed an antioxidant activity of 9.5%. This suggests that this variable depends on flavonoid and condensed tannin contained on the respective seed. These authors showed that thermal treatment differently affected the antioxidant capacity of all varieties under study. Pressure cooking caused a marked decreased of the antioxidant activity on the “Flor de Junio” bean (loss of 71%), whereas cooking on a frying pan affected the “Peruano” bean the most, causing a 92% decrease. In this study, they also observed that canning increased the antioxidant capacity of both varieties. This is attributed to an increased phenolic antioxidant release caused by the double thermal treatment. Rocha-Guzmán et al. [109] measured a higher antioxidant capacity on the “Pinto Saltillo” bean, followed by “Pinto Durango”, “Bayo Victoria” and “Negro 8025”. Raw bean possessed a higher activity when compared to the cooked form. These authors found that “Bayo Victoria” is the most suitable variety for canning as it exhibited the highest values of antioxidant capacity and phenolic compounds after processing. Conversely, “Negro 8025” displayed the highest antioxidant capacity after the cooking process on the open frying pan. In yet another study, Iniestra-González et al. [16] studied the antioxidant capacity of 16 Mexican bean varieties characterized by different grain types: black, pinto, cream or bayo, azufrado, Flor de mayo and white. They reported that the “Flor de Mayo M38” and “Negro Durango” displayed the highest antioxidant capacities and a considerable amount of anti-nutritionals. Thus, beans with black-colored testa and bayo are distinguished by the highest antioxidant activity, whereas “azufrado” showed the lowest levels.

## 6. Micronutrients

### 6.1. Minerals

The common bean is an important source of selenium [8], calcium, iron, phosphorous, magnesium and zinc [35]. The calcium contained in beans has higher bioavailability regarding magnesium or potassium. The average concentrations of minerals are: cupper 18 mg/kg, iron 60 mg/kg, manganese 23 mg/kg, zinc 29 mg/kg and sulfur 234 mg/kg. Pressure cooking and previous maceration affect iron and zinc retention of cooked bean seeds [37]. However, other minerals such as P, K, Ca, S, Cu, Mn, and Zn, are unaffected by the cooking process [110].

A study conducted by Acosta-Gallegos et al. [111] on 25 enhanced Mexican bean genotypes contained calcium within a 0.11–0.63% range, with the “Azufrado Higuera” being the one containing the lowest levels and the “Ejotero 1” being the one with the highest. Iron content was within the 24.8–57.5 ppm range. In this case the “Negro Otomí” bean contained the highest levels and “Flor de Mayo Anita” the lowest. Furthermore, zinc concentration was within the 27.1–41.3 ppm range and the “Pinto Saltillo” bean contained the highest whereas “Canario 60” had the lowest (Table 6). In that work, they also studied 10 native bean harvests from several states in Mexico and the average minerals content was the following: calcium 0.211%, iron 42.39 ppm and zinc 30.83 ppm.

The mineral content in beans is greatly affected by its origin site. Differences among these populations mainly arise from their respective genetic structure that in turn is influenced by the grain selection made by farmers. Espinoza-García et al. [112] analyzed the mineral content on a collection of 67 native bean populations from four regions of the Oaxaca state (Mexico). Intra- and inter-group differences were observed. The populations from the Central Valleys contained higher levels of P, Na, Ca, Zn and Cu, whereas those from the Sierra Norte Mountains were superior in S, K, Fe, Zn and Mn content. Additionally, other materials rich in Fe and Zn correlated with malnutrition and health issues were detected.

Iron deficiency is the most important nutritional disorder worldwide. It is observed as anemia and it affects more than 1600 million people, whereas zinc deficiency is considered the fifth most important risk factor in developing countries. Biofortification is a process by which mineral content may be enhanced in beans. This is achieved by the application of fertilizers to the soil or via foliar [113].

Beans are an iron and zinc source. Although Fe content may be significantly variable among varieties (e.g., white bean contains twice as much as black), a half-cup portion provides almost 10% of the daily recommended consumption. However, most of the iron in legumes is tightly bound to phytates. These impinge on their absorption, thus contributing to deficiencies in countries where legumes are basic foods. Nevertheless, maceration, germination and fermentation have shown to be effective in order to significantly reduce the content of such compounds [8].

Zinc is a catalytic and structural cofactor for a number of enzymes and other proteins. For several years it has been known that deficiency of this phytonutrient cause an increased oxidative stress and consequently an oxidative damage to DNA, proteins and lipids. These results have highlighted that zinc has an important role as an indirect antioxidant and its absence in a diet may contribute to pathological states such as cancer. Nevertheless, the deficiency of this mineral is a world health problem [114]. By supplying a suitable amount of this mineral on the diet may contribute to a normal pregnancy, it helps for the adequate growth and development of the child and it has an impact on immune function and neurobehavioral development. In populations susceptible to zinc deficiency its preventive supplementation decreases the incidence of preterm births, the morbidity caused by childhood diarrhea and acute infections of the upper respiratory pathways, it promotes linear growth and weight gain in infants and young children. It additionally decreases the duration and severity of diarrheic episodes [115]. It has been also observed that deficiencies of this phytonutrient cause damages to DNA in peripheral blood cells in rats [8].

Legumes are a selenium source. It is known that this phytonutrient plays an important role to prevent breast, esophagus and stomach cancers because of its ability to inhibit tumor cell development on mouse. A case study showed that those subjects possessing higher selenium levels in blood were less exposed to the risk of developing prostate cancer [8]. Additionally, some experimental studies have shown that Se has an inhibitory effect on immunodeficiency virus (HIV) in vitro through its effects on glutathion peroxidase and other selenoproteins. Furthermore, some cohort studies have demonstrated that selenium deficiency is correlated to acquired immune deficiency syndrome (AIDS) progression or mortality [116]. This deficiency has been also linked to Keshan disease, a cardiomyopathy mainly affecting children and fertile women that is frequently fatal and it was named after the northeast Chinese province in which it was endemic. Low selenium levels also increase the risk for liver cancer in hepatitis B/C-positive patients, whereas other studies have evidenced Se beneficial effects against the risk for lung, bladder, gastric esophagitis and prostate cancers [117].

### 6.2. Vitamins

Vitamins are organic compounds that cannot be synthesized by humans and therefore must be ingested to prevent metabolic disorders, although vitamin deficiency syndromes such as scurvy, beriberi, and pellagra are now uncommon in Western societies, specific cliniccal subgroups remain in risk, for example, elderly patients are particularly at risk for vitamins B_12_ and D deficiency, alcohol dependent individuals are at risk for folate, B_6_, B_12_ and thiamin deficiency, and hospitalized patients are at risk for deficiencies of folate and other water soluble vitamins [118].

Beans are good source of folate, tocopherols, thiamine, riboflavin, niacin, biotin, and pyridoxaimine [119]. Vitamins content in nine commercial *Phaseolus vulgaris* classes were evaluated by Augustin et al. [120]. On a 100 g dry weight basis, the mean vitamin values of the raw bean samples amounted to the following: thiamin 0.99 mg, riboflavin 0.20 mg, niacin 1.99 mg, vitamin B_6_ 0.49 mg, folic acid 0.30 mg. Retention values of water-soluble vitamins during cooking between bean classes averaged between 70% and 75%. Olanipekun et al. [121] also reported a reduction in the vitamin content in kidney beans seed flour due to the effect of processing. Davey et al. [122] reported that vitamins are lost during thermal processing because they are highly sensitive to oxidation. Table 7 shows the content of raw and cooked pinto beans; the thermal processing decreased the content of the water soluble vitamins, vitamin C was the most affected by the cooking process, this decreased by 87%, followed by thiamine which decreased by 73% [123]. 

**Figure 4:**
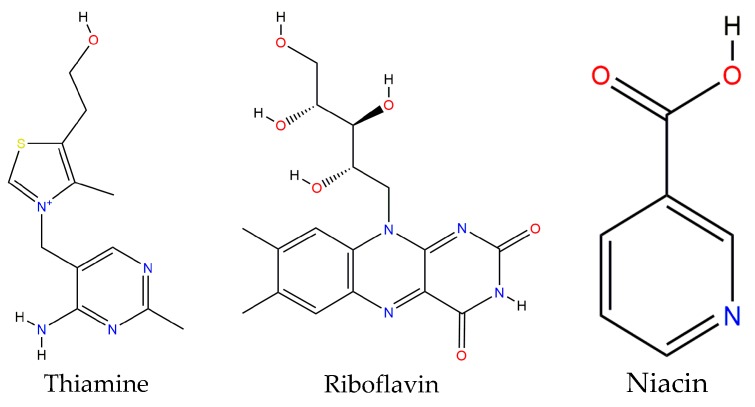

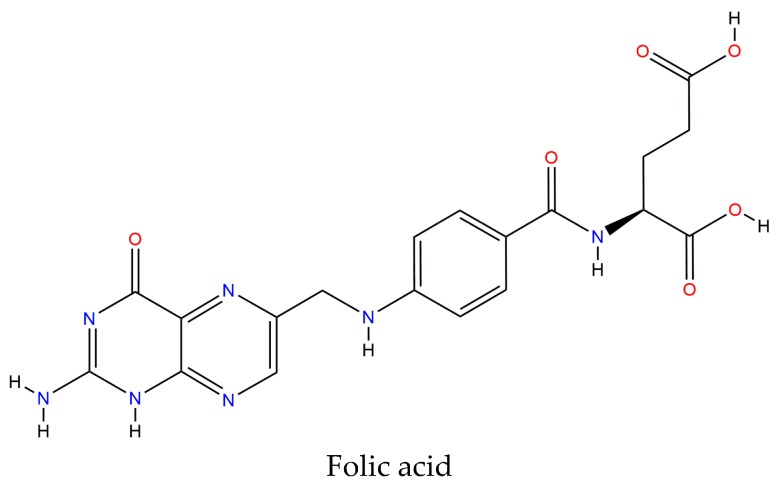
Structures of major vitamins of *P. vulgaris* [46].

Common bean is one of the main sources of vitamins B complex in the diet of the Mexican people, especially thiamine, riboflavin, niacin and folic acid (Table 8) [5] (Figure 4).

Folic acid has been suggested to be a potential nutraceutical. A 100 g portion of common bean can satisfy the daily requirement (0.18–0.4 mg/day) for folic acid for both adults and children [124,125]. Vitamin B_6_ and Vitamin B_12_ have been investigated, usually in conjunction with folic acid, for their role in protecting against cardiovascular disease, especially stroke [126].

Folic acid is needed for healthy blood cell production to prevent anemia in children and adults. Deficiencies of B-complex vitamins lead to megaloblastic anemia, in which the proper maturation of red blood cells is impaired, resulting in fewer red blood cells and release of the large nucleated precursor cells. Folic acid deficiency during pregnancy may lead to neural tube defects, and severe deficiency causes megaloblastic anemia. Common bean can contribute substantial amounts of thiamine to a diet, since a 100 g portion contains close to the recommended dietary allowance for adults (1.2 mg/day) and fully satisfies the daily intake for children (0.9 mg/day). Thiamin deficiency symptoms include fatigue, irritability, weight loss, gastrointestinal disturbances, and cardiovascular complications [123,125]. In addition, dry beans (100 g cooked) contain vitamins B_6_ that account for 13% of the DRI (1.7 mg/day) [123,127]. Other sources of vitamin B_6_ are meat, egg, poultry, legumes and bananas. The total vitamin B_6_ content in fresh pork meat varied from 0.46 to 0.57 mg/100 g, raw chicken egg yolk contained 0.44 mg/100 g and in fluid milk ranged from 0.03 mg to 0.05 mg/100 g [128]. The latter result is lower than that reported in pinto beans [123].

Folic acid is also found in leafy green vegetables (19.9 mg/100 g) and in milk products (2.7 mg/100 g), while dry fruits and cereals are also sources of thiamine (0.45 mg/100 g and 0.17 mg/100 g respectively) [129].

## 7. Conclusions

According to the definition of functional food, the common bean is considered as such because it contains several bioactive compounds that have beneficial impacts on the consumer’s health. There is a great genotype diversity in Mexico and they possess different chemical and physical features. The analyzed evidence confirms that this grain is a rich source of bioactive compounds that positively impact health in several ways. The content of these compounds is highly correlated to the seed coat color and dark-colored or black beans are distinguished by their elevated content of polyphenols, anthocyanins, condensed tannins and flavonoids as quercetin. These confer them with a higher antioxidant capacity. Examples are the Mexican varieties “Negro San Luis”, “Negro Jamapa” and “Negro 8025”, which are the most studied within this category. Nevertheless, bayo and azufrado beans are also prominent because of their high content of prebiotics, e.g., oligosaccharides.

The bean is a food frequently consumed in its cooked form. This thermal treatment significantly impacts on content of bioactive compounds. Contrastingly, this process is necessary to decrease the levels of anti-nutrients such as lectins, phytic acid, tannins, saponins and protease inhibitors that, according to previously obtained evidence, are also potential nutraceutical compounds that may be beneficial to treat cancer, cardiovascular diseases, HIV, diabetes, obesity, neurodegenerative diseases, among others. However, there is a lot of research that is still to be conducted in this regard.

Even though beans are traditionally included in the diet in Mexico and they are a potential functional food contributing to health preservation and disease prevention, a considerable amount of research needs to be conducted in order to characterize the highly diverse genotypes regarding their content of bioactive compounds and their effect on the consumer’s health status. The reviewed information in this work points out the necessity to study wild-type genotypes to know their nutraceutical qualities to be used as valuable resource in enhancement programs and in strategies aimed to encourage its consumption.

## Figures and Tables

**Table 1:** Chemical composition of Mexican varieties of common bean raw.

Variety	Ash (%)	Lipids (%)	Protein (%)	Total Starch (%)	Carbohydrates (%)	Raw Fiber (%)	Humidity (%)
Negro 8025 ^1^	4.20–4.7	1.93–2.0	15.0–23.93	35.27	na	na	8.0
Pinto Durango ^1^	3.96–4.2	1.3–1.56	16.7–22.65	39.84	na	na	10.1
Bayo Victoria ^2^	4.08	0.92	26.35	na	51.51	2.77	10.16
Pinto Saltillo ^2^	3.80	1.71	21.01	na	57.19	1.35	11.58
Negro San Luis ^2^	3.87	0.99	21.68	na	56.28	1.77	11.95

Source: ^1^ [38,39]; ^2^ [40]. Na = not available.

**Table 2:** Saponin content in bean raw cultivar “Negro San Luis”.

Saponins	Concentration (mg/100 g Sample)
Soyasaponin Af (Acetyl A2)	15.67
Deacetyl soyasaponin Af (A2)	3.13
Group A, Total	18.80
Soyasaponin Ba (V) ME	3.81
Soyasaponin αg	11.03
Soyasaponin βg	5.99
Soyasaponin γg	2.65
Group B, Total	23.48
Total soyasaponins	42.28

Source: [45].

**Table 3:** Flavonoid content in raw black bean “Negro San Luis”.

Flavonoids	Concentration (mg/100 g Sample)
Myricetin 3-*O*-glucoside	115.72
Quercetin 4-*O*-galactoside	643.18
Kaempferol 3-*O*-glucoside	6.60

Source: [45].

**Table 4:** Soluble and non-soluble fiber content on the Mexican bean varieties in their cooked and raw forms.

Variety	Soluble Fiber (%)		Non-Soluble Fiber (%)	
	Cooked	Raw	Cooked	Raw
Negro 8025	11.0	1.6	37.5	36.1
Bayo Madero	14.0	0.6	41.0	25.2
Pinto Durango	5.5	0.3	31.1	28.2
Azufrado Higuera	11.0	1.0	31.0	29.3

Source: [38].

**Table 5:** Oligosaccharide content on the varieties of the Mexican common bean.

Bean Variety	Oligosaccharide Content (mg/g)
Negro Durango	53
Negro Sahuatoba	51.9
Negro Altiplano	56.0
Pinto Mestizo	47
Pinto Bayacora	47.2
Pinto Villa	48.3
Bayo Durango	52.3
Bayo Victoria	54.3
Bayo Madero	51.5
Flor de Mayo Bajío	39.0
Flor de Mayo M38	57.6
Mayocoba	51.5
Azufrado Regional87	55.1
Azufrado Peruano 87	54.3
Azufrado Namiquipa	43.1
Perry Marrow	48.1

Source: [16].

**Table 6:** Calcium, iron and zinc content on the seed of 25 enhanced genotypes of the Mexican common bean.

Genotype	Calcium (%)	Iron (ppm)	Zinc (ppm)
Pinto Saltillo	0.19	35	27.1
AFN	0.22	54	34.6
Negro Otomí	0.21	57.5	27.7
Negro Altiplano	0.18	53.2	32.9
Azufrado Namiquipa	0.26	55.2	30.2
Pinto Villa	0.43	33.9	36.2
Flor de Mayo Anita	0.21	24.8	30.3
Flor de Junio Marcela	0.19	35.1	27.8
Rayado Rojo	0.23	36.3	30.6
Negro 8025	0.33	39.7	27.7
Canario 60	0.16	35.7	41.3
Cacahuate Bola	0.2	36.9	29.9
Blanco Español	0.3	53.1	36
Azufrado Higuera	0.11	39.1	34.3
Azufrado 26	0.57	41.1	31.6
Flor de Junio P102	0.2	47.1	29.8
Flor de Junio P114	0.23	46.4	32.1
Flor de Junio P106	0.29	36.5	34.8
A195	0.3	37.8	33.5
Ejotero 1	0.63	39.6	37
Ejotero 7	0.14	43	33.3
Ejotero 22	0.17	53.5	39.1
F. M. Querétaro 501	0.22	41.8	32.6
Pinto Durango	0.17	46	33.4
Flor de Mayo M38	0.14	47.2	28.8

Source: [111].

**Table 7:** Vitamins content of raw and cooked pinto beans.

	Value for 100 g	
	Raw	Cooked *
Vitamin C (total ascorbic acid)	6.30 mg	0.80 mg
Thiamine	0.71 mg	0.19 mg
Riboflavin	0.21 mg	0.06 mg
Niacin	1.17 mg	0.32 mg
Vitamin B_6_ (Pyridoxine)	0.47 mg	0.23 mg
Folate, DFE	0.53 mg	0.17 mg
Vitamin E (alpha-tocopherol)	0.21 mg	0.94 mg
Vitamin E (alpha-tocopherol)	0.21 mg	0.94 mg
Vitamin K (phylloquinone)	5.6 μg	3.5 μg

Source: [123]. * Without addition of salt. DFE = dietary folate equivalents.

**Table 8:** Vitamins content of raw common bean. Celaya, Gto. Mexico.

Vitamin	Content (mg/100 g)
Thiamine	0.9–1.2
Riboflavin	0.14–0.27
Niacin	1.16–2.68
Folic acid (B_9_)	0.17–0.59

Source: [5].

## Abbreviations

AACC	American Association of Cereal Chemists
CE	Catechin Equivalents; DFE, Dietary Folate Equivalents
DRI	Dietary Reference Intake
EC3G	Equivalents to Cyaniding 3-glucoside
GAE	Gallic Acid Equivalent
HAU	Hemagglutinating Unit
TIU	Trypsin Inhibitor Units

## References

[1] Silva-Cristobal L., Osorio-Díaz P., Tovar J., Bello-Pérez L.A. (2010). Chemical composition, carbohydrate digestibility, and antioxidant capacity of cooked black bean, chickpea, and lentil Mexican varieties [Composición química, digestibilidad de carbohidratos, y capacidad antioxidante de variedades mexicanas cocidas de frijol negro, garbanzo, y lenteja]. CyTA J. Food..

[2] Messina V. (2014). Nutritional and health benefits of dried beans. Am. J. Clin. Nutr..

[3] Moreno-Franco B., León-Latre M., Andrés-Esteban E.M., Ordovás J.M., Casasnovas J.A., Peñalvo J.L. (2014). Soluble and insoluble dietary fibre intake and risk factors for cvd and metabolic syndrome in middle-aged adults: The AWHS cohort. Atherosclerosis.

[4] World Health Organization (WHO) Enfermedades no Transmisibles [Non-Transmissible Diseases]. http://www.who.int/mediacentre/factsheets/fs355/es/.

[5] Guzmán M.S.H., Gallegos J.A.A., Muñoz M.D.L.Á.Á., Delgado S.G., Piña G.L. (2002). Calidad alimentariay potencial nutracéutico del frijol (*Phaseolus vulgaris* L.) [Food quality and nutraceutical potential of common bean (*Phaseolus vulgaris* L.)]. Agric. Téc. Méx..

[6] Luthria D.L., Pastor-Corrales M.A. (2006). Phenolic acids content of fifteen dry edible bean (*Phaseolus vulgaris* L.) varieties. J. Food Compost. Anal..

[7] Aparicio-Fernández X., García-Gasca T., Yousef G.G., Lila M.A., González de Mejia E., Loarca-Pina G. (2006). Chemopreventive activity of polyphenolics from black Jamapa bean (*Phaseolus vulgaris* L.) on HeLa and HaCaT cells. J. Agric. Food Chem..

[8] Mudryj A.N., Yu N., Aukema H.M. (2014). Nutritional and health benefits of pulses. Appl. Physiol. Nutr. Metab..

[9] Cardador-Martinez A., Castano-Tostado E., Loarca-Pina G. (2002). Antimutagenic activity of natural phenolic compounds present in the common bean (*Phaseolus vulgaris*) against aflatoxin B 1. Food Addit. Contam..

[10] Hernández-López V.M., Vargas-Vázquez M., Luisa P., Muruaga-Martínez J.S., Hernández-Delgado S., Mayek-Pérez N. (2013). Origen, domesticación y diversificación del frijol común: Avances y perspectivas. Rev. Fitotec. Mex..

[11] Muñoz S. (2010). Frijol, rica Fuente de proteínas. CONABIO. Biodiversitas.

[12] Almanza-Aguilera E., Guzmán-Tovar I., Acosta-Gallegos J.A., Guzmán-Maldonado S.H. Contenido de fitoquímicos en grano de frijol Negro cocido y refrito. Proceedings of the Memoria del Congreso Internacional de Industria y Tecnología Alimentaria [Content of Phytochemicals in Beans of Cooked and Refried BLACK Beans. Report of the International Congress of Industry and Food Technology].

[13] Juárez-López B.A., Aparicio-Fernández X., Nevárez-Moorillón G.V., Ortega-Rivas E. (2012). Polyphenolics Concentration and Antiradical Capacity of Common Bean Varieties (*Phaseolus vulgaris* L.) after Thermal Treatment. Food Science and Food Biotechnology Essentials: A Contemporary Perspective.

[14] Espinosa-Alonso L.G., Lygin A., Widholm J.M., Valverde M.E., Paredes-Lopez O. (2006). Polyphenols in wild and weedy Mexican common beans (*Phaseolus vulgaris* L.). J. Agric. Food Chem..

[15] Martinez P.A.H., Naranjo F.A., Nugaray A.J. Antocianinas, flavonoides y ácidos fenólicos presentes en frijol negro Querétaro y Mayocoba y su estabilidad durante el cocimiento industrial [Anthocyanins, flavonoids and phenolic acids present in Querétaro and Mayocoba black beans and their stability during industrial cooking]. Proceedings of the X Congreso Nacional de Biotecnología y Bioingeniería [X National Congress of Biotechnology and Bioengineering].

[16] Iniestra-González J.J., Ibarra-Pérez F.J., Gallegos-Infante J.A., Rocha-Guzmán N.E., González-Laredo R.F. (2005). Factores antinutricios y actividad antioxidante en variedades mejoradas de frijol común (*Phaseolus vulgaris*) [Antinutritional factors and antioxidant activity in improved varieteies of common bean (*Phaseolus vulgaris*)]. Agrociencia.

[17] González de Mejía E.G., Guzmán-Maldonado S.H., Acosta-Gallegos J.A., Reynoso-Camacho R., Ramírez-Rodríguez E., Pons-Hernández J.L., González-Chavira M.M., Castellanos J.Z., Kelly J.D. (2003). Effect of cultivar and growing location on the trypsin inhibitors, tannins, and lectins of common beans (*Phaseolus vulgaris* L.) grown in the semiarid highlands of Mexico. J. Agric. Food Chem..

[18] Reynoso Camacho R., del Carmen Ríos Ugalde M., Torres Pacheco I., Acosta Gallegos J.A., Palomino Salinas A.C., Ramos Gómez M., González Jasso E., Horacio Guzmán Y.S.H. (2007). El consumo de frijol común (*Phaseolus vulgaris* L.) y su efecto sobre el cáncer de colon en ratas Sprague-Dawley [Common bean (*Phaseolus Vulgaris* L.) consumption and its effects on colon cancer in Sprague–Dawley rats]. Agric. Téc. Méx..

[19] Raya-Pérez J.C., Gutiérrez-Benicio G.M., Pimentel J.G.R., Prieto J.C., Aguirre-Mancilla C.L. (2014). Caracterización de proteínas y contenido mineral de dos variedades nativas de frijol de México [Characterization of proteins and mineral content of two bean landraces from Mexico]. Agron. Mesoam..

[20] Hasler C.M. (2002). Functional foods: Benefits, concerns and challenges—A position paper from the American Council on Science and Health. J. Nutr..

[21] Henry C.J. (2010). Functional Foods. Eur. J. Clin. Nutr..

[22] Drago S.M.E., López M.L., Espuñes T.D.R. (2006). Componentes bioactivos de alimentos funcionales de origen vegetal [Bioactive Components of Functional Foods from Vegetable Origin]. Rev. Mex. Cienc. Farm..

[23] Health Canada (2000). Standards of Evidence for Evaluating Foods with Health Claims.

[24] Consensus Document (1999). Scientific concepts of functional foods in Europe consensus document. Br. J. Nutr..

[25] Serafini M., Stanzione A., Foddai S. (2012). Functional foods: Traditional use and European legislation. Int. J. Food Sci. Nutr..

[26] Nicoletti M. (2012). Nutraceuticals and botanicals: Overview and perspectives. Int. J. Food Sci. Nutr..

[27] Hernandez F.A., Gil F., Ramesh C.G. (2016). Nutraceutical an adverse outcome pathway. Nutraceuticals: Efficacy, Safety and Toxicity.

[28] Biesalski H.K., Dragsted L.O., Elmadfa I., Grossklaus R., Müller M., Schrenk D., Walter P., Weber P. (2009). Bioactive compounds: Definition and assessment of activity. Nutrition.

[29] Nasri H., Baradaran A., Shirzad H., Rafieian-Kopaei M. (2014). New concepts in nutraceuticals as alternative for pharmaceuticals. Int. J. Prev. Med..

[30] Dureja H., Kaushik D., Kumar V. (2003). Developments in nutraceuticals. Indian J. Pharmacol..

[31] Palthur M.P., Palthur S.S., Chitta S.K. (2010). Nutraceuticals: A conceptual definition. Int. J. Pharm Pharm. Sci..

[32] Sarin R., Sharma M., Singh R., Kumar S. (2012). Nutraceuticals: A review. Int. Res. J. Pharm..

[33] Gálvez A., Salinas G. (2015). El papel del frijol en la salud nutrimental de la población mexicana [The role of beans in the nutritional health of the Mexican population]. Rev. Digit. Univ..

[34] Granito M., Guinand J., Pérez D., Pérez S. (2009). Valor nutricional y propiedades funcionales de *Phaseolus vulgaris* procesada: Un ingrediente potencial para alimentos [Nutritive value and functional properties of processed *Phaseolus vulgaris*: A potential food ingredient]. Interciencia.

[35] Ulloa J.A., Rosas U.P., Ramírez R.J.C., Rangel U.B.E. (2011). El frijol (*Phaseolus vulgaris*): Su importancia nutricional y como fuente de fitoquímicos [Beans (*Phaseolus vulgaris*): Their nutritional importance and source of phytochemicals]. Rev. Fuente.

[36] Mederos Y. (2006). Indicadores de la calidad en el grano de frijol (*Phaseolus vulgaris* L.) [Quality indicators in bean (*Phaseolus vulgaris* L.)]. Cultiv. Trop..

[37] Suárez-Martínez S.E., Ferriz-Martínez R.A., Campos-Vega R., Elton-Puente J.E., de la Torre Carbot K., García-Gasca T. (2016). Bean seeds: Leading nutraceutical source for human health. CyTA-J. Food..

[38] Campos-Vega R., Reynoso-Camacho R., Pedraza-Aboytes G., Acosta-Gallegos J.A., Guzman-Maldonado S.H., Paredes-Lopez O., Oomah B.D., Loarca-Piña G. (2009). Chemical composition and in vitro polysaccharide fermentation of different beans (*Phaseolus vulgaris* L.). J. Food Sci..

[39] Ovando-Martínez M., Osorio-Díaz P., Whitney K., Bello-Pérez L.A., Simsek S. (2011). Effect of the cooking on physicochemical and starch digestibility properties of two varieties of common bean (*Phaseolus vulgaris* L.) grown under different water regimes. Food Chem..

[40] Aguirre S.E.A., Gómez-Aldapa C.A. Evaluación de las características fisicoquímicas en la especie de frijol *Phaseolus vulgaris* de las variedades; Pinto Saltillo, Bayo Victoria y Negro San Luis [Evaluation of physicochemical characteristics in *Phaseolus vulgaris* beans species; Pinto Saltillo, Bayo Victoria and Black San Luis]. Proceedings of the XII Congreso Nacional de Ciencia y Tecnología de Alimentos [XII National Congress of Food Science and Technology].

[41] Sancho R.A.S., Pavan V., Pastore G.M. (2015). Effect of in vitro digestion on bioactive compounds and antioxidant activity of common bean seed coats. Food Res. Int..

[42] Ramírez-Jiménez A.K., Reynoso-Camacho R., Tejero M.E., León-Galván F., Loarca-Piña G. (2015). Potential role of bioactive compounds of *Phaseolus vulgaris* L. on lipid-lowering mechanisms. Food Res. Int..

[43] Ávalos G.A., Pérez-Urria C.E. (2009). Metabolismo secundario de plantas [Secondary plant metabolism]. Reduca (Biología). Ser. Fisiol. Veg..

[44] Elizalde A.D.D., Porrilla Y.P., Chaparro D.C.C. (2009). Factores antinutricionales en semillas [Antinutritional factors in eatable sedes]. Biotecnol. Sect. Agropecu. Agroind..

[45] Guajardo-Flores D., García-Patiño M., Serna-Guerrero D., Gutiérrez-Uribe J.A., Serna-Saldívar S.O. (2012). Characterization and quantification of saponins and flavonoids in sprouts, seed coats and cotyledons of germinated black beans. Food Chem..

[46] PubChem Compound, NCBI Database. http://www.ncbi.nlm.nih.gov/pccompound.

[47] Shi J., Arunasalam K., Yeung D., Kakuda Y., Mittal G., Jiang Y. (2004). Saponins from edible legumes: Chemistry, processing, and health benefits. J. Med. Food.

[48] Sivaci A., Duman S. (2014). Evaluation of seasonal antioxidant activity and total phenolic compounds in stems and leaves of some almond (*Prunus amygdalus* L.) varieties. Biol. Res..

[49] Huber K., Brigide P., Bretas E.B., Canniatti-Brazaca S.G. (2016). Phenolic Acid, Flavonoids and Antioxidant Activity of Common Brown Beans (*Phaseolus vulgaris* L.) Before and After Cooking. J. Nutr. Food Sci..

[50] Akillioglu H.G., Karakaya S. (2010). Changes in total phenols, total flavonoids, and antioxidant activities of common beans and pinto beans after soaking, cooking, and in vitro digestion process. Food Sci. Biotechnol..

[51] González de Mejía E.G., Castaño-Tostado E., Loarca-Piña G. (1999). Antimutagenic effects of natural phenolic compounds in beans. Mutat. Res. Genet. Toxicol. Environ. Mutagen..

[52] Boateng J., Verghese M., Walker L.T., Ogutu S. (2008). Effect of processing on antioxidant contents in selected dry beans (*Phaseolus* spp. L.). Food sci. Technol..

[53] Orak H.H., Karamać M., Orak A., Amarowicz R. (2016). Antioxidant Potential and Phenolic Compounds of Some Widely Consumed Turkish White Bean (*Phaseolus vulgaris* L.) Varieties. Pol. J. Food Nutr. Sci..

[54] Mujica M.V., Granito M., Soto N. (2012). Variación de los compuestos fenólicos de *Phaseolus vulgaris* L. durante el almacenamiento y su relación con el endurecimiento [Variation of phenolic compounds of *Phaseolus vulgaris* L. during storage and their relationship to hardening]. Bioagro.

[55] Herrera Hernández M.G., Acosta Gallegos J.A., Salinas Pérez R.A., Bernardo Casas A.M., Guzmán Maldonado Y.S.H. (2014). Componentes relacionados con la salud en semillas de frijol de plantas crecidas bajo riego y estrés hídrico terminal [Health-related components in plant bean seeds grown under irrigation and terminal drought stress]. Rev. Mex. Cienc. Agrícolas.

[56] Aparicio-Fernandez X., Yousef G.G., Loarca-Pina G., de Mejia E., Lila M.A. (2005). Characterization of polyphenolics in the seed coat of Black Jamapa bean (*Phaseolus vulgaris* L.). J. Agric. Food Chem..

[57] Díaz-Batalla L., Widholm J.M., Fahey G.C., Castaño-Tostado E., Paredes-López O. (2006). Chemical components with health implications in wild and cultivated Mexican common bean seeds (*Phaseolus vulgaris* L.). J. Agric. Food Chem..

[58] Li Y., Yao J., Han C., Yang J., Chaudhry M.T., Wang S., Liu H., Yin Y. (2016). Quercetin, inflammation and immunity. Nutrients.

[59] Jacques P.F., Cassidy A., Rogers G., Peterson J.J., Meigs J.B., Dwyer J.T. (2013). Higher dietary flavonol intake is associated with lower incidence of type 2 diabetes. J. Nutr..

[60] McCullough M.L., Peterson J.J., Patel R., Jacques P.F., Shah R., Dwyer J.T. (2012). Flavonoid intake and cardiovascular disease mortality in a prospective cohort of US adults. Am. J. Clin. Nutr..

[61] Nettleton J.A., Harnack L.J., Scrafford C.G., Mink P.J., Barraj L.M., Jacobs D.R. (2006). Dietary flavonoids and flavonoid-rich foods are not associated with risk of type 2 diabetes in postmenopausal women. J. Nutr..

[62] Morimoto Y., Maskarinec G., Park S.Y., Ettienne R., Matsuno R.K., Long C., Steffen A.D., Henderson B.E., Kolonel L.N., Le Marchand L. (2014). Dietaryisoflavone intake is not statistically significantly associated with breast cancer risk in the Multiethnic Cohort. Br. J. Nutr..

[63] Chang H., Xie Q., Zhang Q., Peng X., Zhu J., Mi M. (2013). Flavonoids, flavonoid subclasses and breast cancer risk: A meta-analysis of epidemiologic studies. PLoS ONE.

[64] Scholz S., Williamson G. (2007). Interactions affecting the bioavailability of dietary polyphenols in vivo. Int. J. Vitam. Nutr. Res..

[65] Gaine P.C., Balentine D.A., Erdman J.W., Dwyer J.T., Ellwood K.C., Hu F.B., Russell R.M. (2013). Are dietary bioactives ready for recommended intakes?. Adv. Nutr..

[66] Jun S., Shin S., Joung H. (2016). Estimation of dietary flavonoid intake and major food sources of Korean adults. Br. J. Nutr..

[67] Sebastian R.S., Enns C.W., Goldman J.D., Martin C.L., Steinfeldt L.C., Murayi T., Moshfegh A.J. (2015). A new database facilitates characterization of flavonoid intake, sources, and positive associations with diet quality among US adults. J. Nutr..

[68] Peterson J.J., Dwyer J.T., Jacques P.F., McCullough M.L. (2015). Improving the estimation of flavonoid intake for study of health outcomes. Nutr. Rev..

[69] Egert S., Wolffram S., Bosy-Westphal A., Boesch-Saadatmandi C., Wagner A.E., Frank J., Rimbach G., Mueller M.J. (2008). Daily quercetin supplementation dose-dependently increases plasma quercetin concentrations in healthy humans. J. Nutr..

[70] Cureton K.J., Tomporowski P.D., Singhal A., Pasley J.D., Bigelman K.A., Lambourne K., Trilk J.L., McCully K.K., Arnaud M.J., Zhao Q. (2009). Dietary quercetin supplementation is not ergogenic in untrained men. J. Appl. Physiol..

[71] Hertog M.G., Hollman P.C., Katan M.B. (1992). Content of potentially anticarcinogenic flavonoids of 28 vegetables and 9 fruits commonly consumed in the Netherlands. J. Agric. Food Chem..

[72] Guzmán-Maldonado S.H., Marín-Jarillo A., Castellanos J.Z., De Mejía E.G., Acosta-Gallegosc J.A. (1996). Relationship between physical and chemical characteristics and susceptibility to Zabrotes subfasciatus (Boh.)(Coleoptera: Bruchidae) and Acanthoscelides obtectus (Say) in common bean (*Phaseolus vulgaris* L.) varieties. J. Stored Prod. Res..

[73] Díaz A.M., Caldas G.V., Blair M.W. (2010). Concentrations of condensed tannins and anthocyanins in common bean seed coats. Food Res. Int..

[74] Schofield P., Mbugua D.M., Pell A.N. (2001). Analysis of condensed tannins: A review. Anim. Feed Sci. Technol..

[75] Takeoka G.R., Dao L.T., Full G.H., Wong R.Y., Harden L.A., Edwards R.H., Berrios S. (1997). Characterization of black bean (*Phaseolus vulgaris* L.) anthocyanins. J. Agric. Food Chem..

[76] Salinas-Moreno Y., Rojas-Herrera L., Sosa-Montes E., Pérez-Herrera P. (2005). Composición de Antocianinas en Variedades de Firijol Negro (*Phaseolus vulgaris* L.) Cultivadas en México [Anthocyanin composition in black bean (*Phaseolus vulgaris* L.) varieties grown in México]. Agrociencia.

[77] Lee J. (2016). Rosaceae products: Anthocyanin quality and comparisons between dietary supplements and foods. NFS J..

[78] Wallace C.T., Giusti M.M. (2015). Anthocyanins. Nutrient information. Adv. Nutr..

[79] Lima J.E., Sampaio A.L.F., Henriques M.M.O., Barja–Fidalgo C. (1999). Lymphocyte activation and cytokine production by *Pisum sativum* agglutinin (PSA) in vivo and in vitro. Immunopharmacology.

[80] Messina M.J. (1999). Legumes and soybeans: Overview of their nutritional profiles and health effects. Am. J. Clin. Nutr..

[81] Setchell K.D., Radd S. (2000). Soy and other legumes: Bean around a long time but are they the superfoods of the millennium and what are the safety issues for their constituent phytoestrogens?. Asia Pac. J. Clin. Nutr..

[82] Xu B., Chang S.K. (2008). Total phenolics, phenolic acids, isoflavones, and anthocyanins and antioxidant properties of yellow and black soybeans as affected by thermal processing. J. Agric. Food Chem..

[83] Rauter A.P., Dias C., Martins A., Branco I., Neng N.R., Nogueira J.M., Goulart M., Silva F.M.V., Justino J., Trevitt C. (2012). Non-toxic Salvia sclareoides Brot. extracts as a source of functional food ingredients: Phenolic profile, antioxidant activity and prion binding properties. Food Chem..

[84] Tosh S.M., Yada S. (2010). Dietary fibers in pulse seeds and fractions: Characterization, functional attributes, and applications. Food Res. Int..

[85] Perry J.R., Ying W. (2016). A review of physiological effects of soluble and insoluble dietary fibers. J. Nutr. Food Sci..

[86] Fujii H., Iwase M., Ohkuma T., Ogata-Kaizu S., Ide H., Kikuchi Y., Idewaki Y., Joudai T., Hirakawa Y., Uchida K. (2013). Impact of dietary fiber intake on glycemic control, cardiovascular risk factors and chronic kidney disease in Japanese patients with type 2 diabetes mellitus: The Fukuoka Diabetes Registry. Nutr. J..

[87] Huang T., Zhang X. (2015). Dietary Fiber Intake and Mortality from All Causes, Cardiovascular Disease, Cancer, Infectious Diseases and Others: A Meta-Analysis of 42 Prospective Cohort Studies with 1,752,848 Participants. N. Am. J. Med. Sci..

[88] Tungland B., Meyer D. (2002). Non-digestible oligo-and polysaccharides (dietary fiber): Their physiology and role in human health and food. Compr. Rev. Food Sci. Food Saf..

[89] Brown K.H., Rivera J.A., Bhutta Z., Gibson R.S., King J.C., Lonnerdal B., Ruel M.T., Sandtröm B., Wasantwisut E., Hotz C. (2004). International Zinc Nutrition Consultative Group (IZiNCG) technical document# 1. Assessment of the risk of zinc deficiency in populations and options for its control. Food Nutr. Bull..

[90] Lião L.M., Alves Filho E.G., Silva L.M.A., Choze R., Alcantara G.B., Bassinello P.Z., Renou J.-P., Belton P.S., Webb G.A. (2011). Quantification of oligosaccharides from common beans by HR-MAS NMR. Magnetic Resonance in Food Science. An Exciting Future.

[91] López-Hernández G., Herrera-Hernández M.G., Figueroa-Gonzales J.J., Guzmán-Maldonado H., Acosta-Gallegos J.A. Contenido de fitoquímicos en diferentes marcas frijol bayo enlatado [Content of phytochemicals in different brands of canned bayo bean]. Proceedings of the XII Congreso Nacional De Ciencia Y Tecnología De Alimentos [XII National Congress of Food Science and Technology].

[92] Miranda-Villa P.P., Marrugo-Ligardo Y.A., Montero-Castillo P.M. (2013). Caracterización Funcional del Almidón de Fríjol Zaragoza (*Phaseolus Lunatus* L.) y Cuantificación de su Almidón Resistente [Functional characterization of bean Zaragoza starch (*Phaseolus Lunatus* L.) and quantification of the resistant starch]. Tecno Lógicas.

[93] Osorio-Díaz P., Bello-Pérez L.A., Sáyago-Ayerdi S.G., Benítez-Reyes M.D.P., Tovar J., Paredes-López O. (2003). Effect of processing and storage time on in vitro digestibility and resistant starch content of two bean (*Phaseolus vulgaris* L) varieties. J. Sci. Food Agric..

[94] Osorio-Díaz P., Tovar J., Paredes-López O., Acosta-Gallegos J.A., Bello-Pérez L.A. (2005). Chemical composition and in vitro starch bioavailability of *Phaseolus vulgaris* (L) cv Mayocoba. J. Sci Food Agric..

[95] Kasote D.M., Nilegaonkar S.S., Agte V.V. (2014). Effect of different processing methods on resistant starch content and in vitro starch digestibility of some common Indian pulses. J. Sci. Ind. Res..

[96] Chan C.K., Ransom R.C., Longaker M.T. (2016). Lectins bring benefits to bones. eLife.

[97] Pervin M., Koyama Y., Isemura M., Nakamura Y. (2015). Plant lectins in therapeutic and diagnostic cancer research. Int. J. Plant Biol. Res..

[98] Kumar K.K., Chandra K.L.P., Sumanthi J., Reddy G.S., Shekar P.C., Reddy B.V.R. (2012). Biological role of lectins: A review. J. Orofac. Sci..

[99] Mendoza W., Gandolfo L., Ponce L., Novello J., Marangoni S. (2007). Estudios estructura y función de una lectina aislada de semillas de Caesalpinia spinosa kuntze (tara) [Structure and function studies of a lectin from Caesalpinia spinosa kuntze (tara) sedes]. Idesia.

[100] Akkouh O., Ng T.B., Singh S.S., Yin C., Dan X., Chan Y.S., Pan W., Cheung R.C. (2015). Lectins with anti-HIV activity: A review. Molecules.

[101] Roy F., Boye J.I., Simpson B.K. (2010). Bioactive proteins and peptides in pulse crops: Pea, chickpea and lentil. Food Res. Int..

[102] Campos-Vega R., Loarca-Piña G., Oomah B.D. (2010). Minor components of pulses and their potential impact on human health. Food Res. Int..

[103] Trowbridge I.S. (1973). Mitogenic properties of pea lectin and its chemical derivatives. Proc. Natl. Acad. Sci. USA.

[104] Paredes-Lopez O., Schevenin M.L., Guevara-Lara F., Barradas I. (1989). Thermal inactivation of haemagglutinating activity of normal and genetically-improved common bean varieties: A kinetic approach. Food Chem..

[105] Coffey D.G., Uebersax M.A., Hosfield G.L., Bennink M.R. (1993). Thermal extrusion and alkali processing of dry beans (*Phaseolus vulgaris* L.). J. Food Process. Preserv..

[106] Guillamon E., Pedrosa M.M., Burbano C., Cuadrado C., de Cortes Sanchez M., Muzquiz M. (2008). The trypsin inhibitors present in seed of different grain legume species and cultivar. Food Chem..

[107] Wang N., Hatcher D.W., Tyler R.T., Toews R., Gawalko E.J. (2010). Effect of cooking on the composition of beans (*Phaseolus vulgaris* L.) and chickpeas (*Cicer arietinum* L.). Food Res. Int..

[108] Petry N., Egli I., Campion B., Nielsen E., Hurrell R. (2013). Genetic reduction of phytate in common bean (*Phaseolus vulgaris* L.) seeds increases iron absorption in young women. J. Nutr..

[109] Rocha-Guzman N.E., Gallegos-Infante J.A., González-Laredo R.F., Cardoza-Cervantes V., Reynoso-Camacho R., Ramos-Gómez M., Garcia-Gasca T., De Anda S.A. (2013). Evaluation of culinary quality and antioxidant capacity for Mexican common beans (*Phaseolus vulgaris* L.) canned in pilot plant. Int. Food Res. J..

[110] Brigide P., Canniatt-Brazaca S.G., Silva M.O. (2014). Nutritional characteristics of biofortified common beans. Food Sci. Technol..

[111] Acosta-Gallegos J.A., Mendoza-Hernandez F.M., Guzman-Maldonado S.H., Hernandez J.Y., Herrera M.D.Y. (2016). Contenido de proteína y minerales en la semilla de frijol silvestre y domesticado. [Protein and mineral content in the seed of wild and domesticated common bean] Revista Mexicana de Ciencias Agrícolas. Pub. Espec..

[112] Espinoza-García N., Martínez-Martínez R., Chávez-Servia J.L., Vera-Guzmán A.M., Carrillo-Rodríguez J.C., Heredia-García E., Velasco-Velasco V.A. (2016). Contenido de minerales en semilla de poblaciones nativas de frijol común (*Phaseolus vulgaris* L.) [Mineral content in seeds of native populations of common bean (*Phaseolus vulgaris* L.)]. Rev. Fitotec. Mex..

[113] Guillén-Molina M., Márquez-Quiroz C., de la Cruz-Lázaro E., Velázquez-Martínez J.R., Parra J.M.S., Carrillo M.G., Vidal J.A.O. (2016). Biofortificación de frijol caupí (*Vigna unguiculata* L. Walp) con hierro y zinc [Biofortification of cowpea (*Vigna unguiculata* L. Walp.) with iron and zinc]. Rev. Mex. Cienc. Agrícolas.

[114] Eide D.J. (2011). The oxidative stress of zinc deficiency. Metallomics.

[115] Wessells K.R., Brown K.H. (2012). Estimating the global prevalence of zinc deficiency: Results based on zinc availability in national food supplies and the prevalence of stunting. PLoS ONE.

[116] Stone C.A., Kawai K., Kupka R., Fawzi W.W. (2010). Role of selenium in HIV infection. Nutr. Rev..

[117] Rayman M.P. (2008). Food-chain selenium and human health: Emphasis on intake. Br. J. Nutr..

[118] Fairfield K.M., Fletcher R.H. (2002). Vitamins for chronic disease prevention in adults: Scientific review. JAMA.

[119] Hayat I., Ahmad A., Masud T., Ahmed A., Bashir S. (2014). Nutritional and health perspectives of beans (*Phaseolus vulgaris* L.): An overview. Crit. Rev. Food Sci. Nutr..

[120] AuguStin J., Beck C.B., Kalbfleish G., Kagel L.C., Matthews R.H. (1981). Variation in the vitamin and mineral content of raw and cooked commercial *Phaseolus vulgaris* classes. J. Food Sci..

[121] Olanipekun O.T., Omenna E.C., Olapade O.A., Suleiman P., Omodara O.G. (2015). Effect of boiling and roasting on the nutrient composition of kidney beans seed flour. J. Food Sci..

[122] Davey J.S., Rickman J.C., Barret D.M., Bruhn C.M. (2000). Nutritional Comparison of fresh and frozen fruits. Sci. Food Agric..

[123] Câmara C.R., Urrea C.A., Schlegel V. (2013). Pinto beans (*Phaseolus vulgaris* L.) as a functional food: Implications on human health. Agriculture.

[124] Guzmán-Maldonado S.H., Paredes-Lopez O., Mazza G. (1998). Functional products of plants indigenous to Latin America: Amaranth, quinoa, common beans, and botanicals. Functional Foods. Biochemical and Processing Aspects.

[125] Centers for Disease Control (1992). Recommendations for the use of folic acid to reduce the number of cases of spina bifida and other neural tube defects. MMWR Recomm. Rep..

[126] Ryan-Harshman M., Aldoori W. (2005). Health benefits of selected vitamins. Can. Fam. Physician.

[127] U.S. Department of Health Human Services Nutrient Recommendations: Dietary Reference Intakes (DRI). https://ods.od.nih.gov/Health_Information/Dietary_Reference_Intakes.aspx.

[128] Ollilainen V.M. (1999). HPLC analysis of vitamin B6 in foods. Agric. Food Sci. Finl..

[129] Agte V., Tarwadi K., Mengale S., Hinge A., Chiplonkar S. (2002). Vitamin profile of cooked foods: How healthy is the practice of ready-to-eat foods?. Int. J. Food Sci. Nutr..

